# Stimuli-Induced Architectural Transition as a Tool for Controlling the Enzymatic Degradability of Polymeric Micelles

**DOI:** 10.1021/acspolymersau.2c00023

**Published:** 2022-07-27

**Authors:** Gadi Slor, Shahar Tevet, Roey J. Amir

**Affiliations:** † Department of Organic Chemistry, School of Chemistry, Faculty of Exact Sciences, Tel-Aviv University, Tel-Aviv 6997801, Israel; ‡ Tel-Aviv University Center for Nanoscience and Nanotechnology, Tel-Aviv University, Tel-Aviv 6997801, Israel; § ADAMA Center for Novel Delivery Systems in Crop Protection, Tel-Aviv University, Tel-Aviv 6997801, Israel; ∥ The Center for Physics and Chemistry of Living Systems, Tel-Aviv University, Tel-Aviv 6997801, Israel

**Keywords:** block copolymers, dendrimers, enzyme-responsive nanocarriers, micelles, polymeric amphiphiles, polymeric micelles

## Abstract

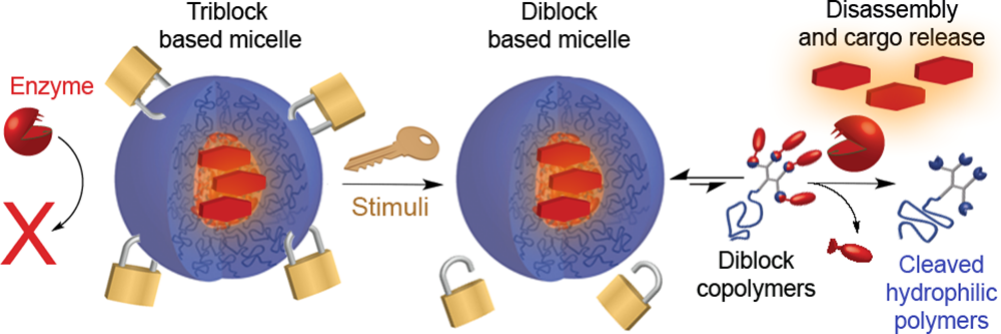

Enzyme-responsive polymeric micelles hold great potential as drug delivery systems due to the overexpression of disease-associated enzymes. To achieve selective and efficient delivery of their therapeutic cargo, micelles need to be highly stable and yet disassemble when encountering their activating enzyme at the target site. However, increased micellar stability is accompanied by a drastic decrease in enzymatic degradability. The need to balance between stability and enzymatic degradation has severely limited the therapeutic applicability of enzyme-responsive nanocarriers. Here, we report a general modular approach for designing stable enzyme-responsive micelles whose enzymatic degradation can be enhanced on demand. The control over their response to the activating enzyme is achieved by stimuli-induced splitting of triblock amphiphiles into two identical diblock amphiphiles, which have the same hydrophilic–lipophilic balance as the parent amphiphile. This architectural transition drastically affects the micelle–unimer equilibrium and therefore increases the sensitivity of the micelles toward enzymatic degradation. As a proof of concept, we designed UV- and reduction-activated splitting mechanisms, demonstrating the ability to use architectural transition as a tool for tuning amphiphile–protein interactions, providing a general solution toward overcoming the stability–degradability barrier for enzyme-responsive nanocarriers.

## Introduction

Polymeric micelles have great potential to serve as smart drug delivery systems (DDSs).^1−4^ To reach their target tissue without premature release of their therapeutic cargo, micelles must be highly stable toward dilution and nonspecific interactions with serum proteins and endothelial cells. At the same time, micelles must also disassemble once they reach their target site to release their therapeutic cargo.^5−9^ Furthermore, micellar degradation and disassembly are crucial for traceless clearance of the DDSs from the body after delivering their payload.^10^ The high specificity and overexpression of disease-associated enzymes in diseased tissues make enzymes highly promising stimuli for triggering the selective release of drugs from micellar nanocarriers.^11,12^ Unlike stimuli-responsive micelles that respond to dimensionless stimuli such as light^13,14^ or temperature,^15^ enzymatically degradable micelles show a reverse correlation between micellar stability and their responsiveness to the activating enzymes.^16^ Once micellar stability reaches a certain threshold, they become unreactive toward the activating enzyme. The challenge to balance between stability and degradability is one of the key limitations of such nanocarriers. In previous reports, the authors and others found that decreasing the overall molecular weight of polymeric amphiphiles, while preserving their hydrophilic-to-lipophilic balance (HLB), significantly reduces micellar stability toward enzymatic degradation.^17−19^ Based on these findings, herein, we set to develop a general strategy to overcome the stability–responsiveness barrier. Our approach is based on using stimuli-induced architectural transition of hydrophobic–hydrophilic–hydrophobic (B-A–B) triblock copolymer (TBC) amphiphiles to hydrophobic–hydrophilic (B-A′) diblock copolymer (DBC) amphiphiles as a tool for enhancing their enzymatic degradability.

**Figure 1:**
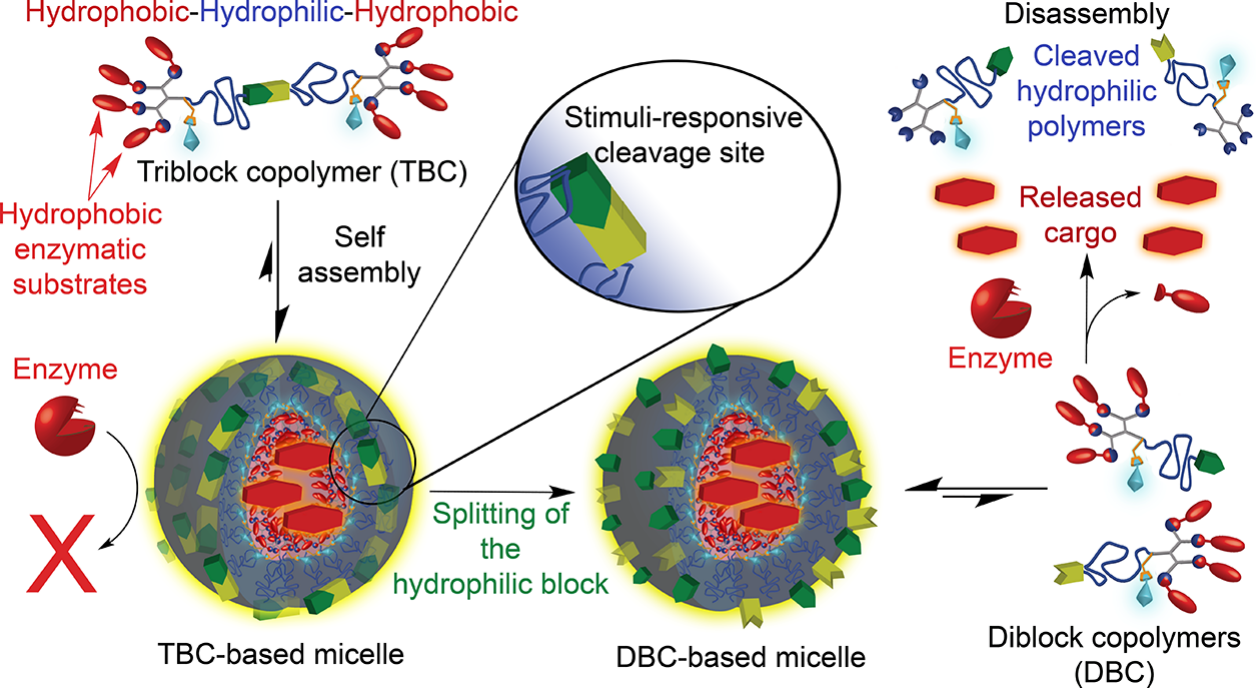
Schematic presentation of splittable TBC amphiphiles and their self-assembly into stable flower-like micelles. Activation of the trigger at the center of the hydrophilic block leads to the splitting of the TBC into two amphiphilic DBCs, which due to the increase in the unimer–micelle exchange rate can be enzymatically degraded into hydrophilic polymers, leading to complete disassembly and release of the encapsulated cargo. The amphiphiles are fluorescently labeled and show red-shifted emission at their assembled state due to close packing of the dyes.

**Scheme 1:**
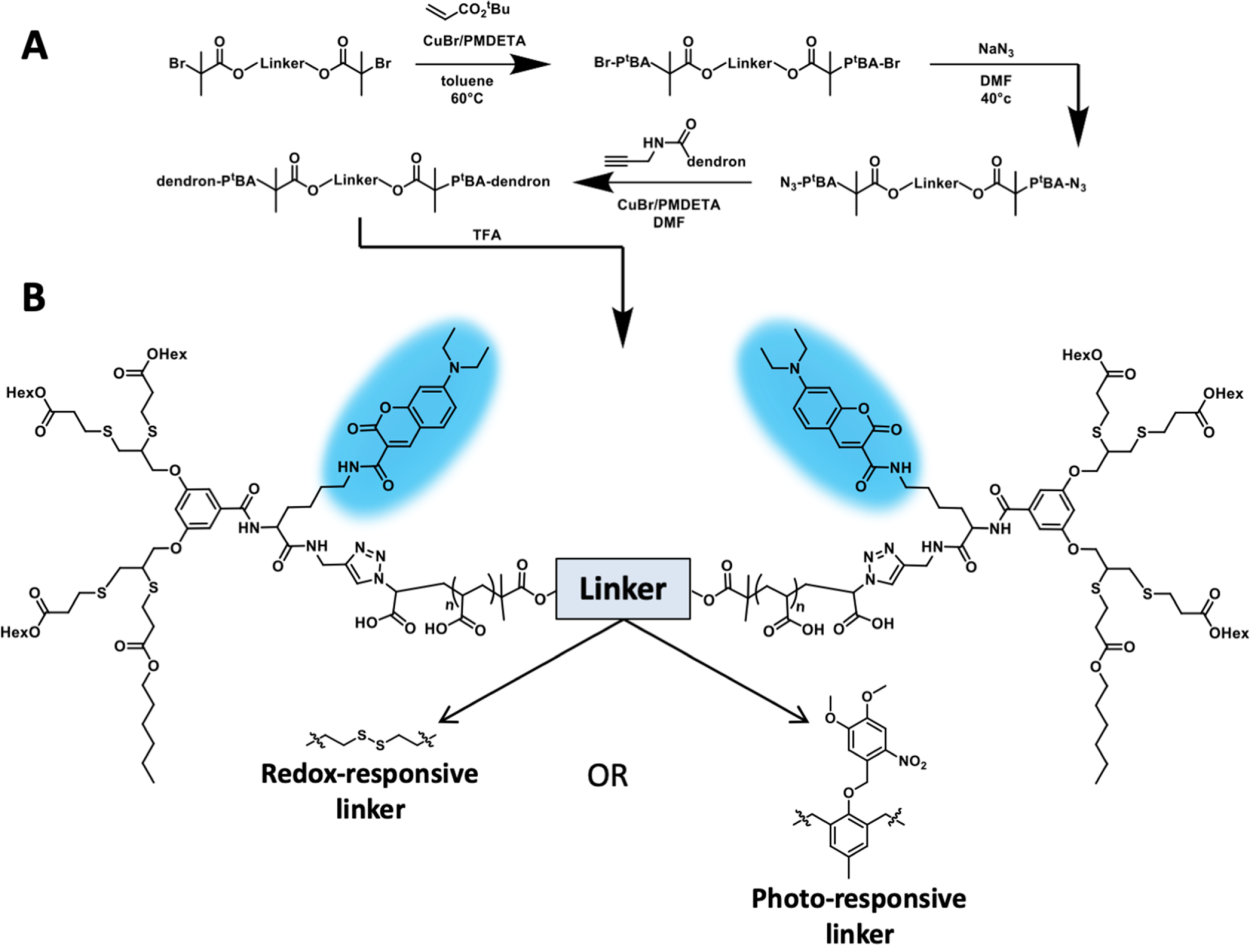
(A) Synthetic Pathway for the Preparation of Enzyme-Responsive TBCs with a Cleavable Linker (Redox- or UV-Responsive) at the Center of Their Hydrophilic A Block; (B) General Structure of the TBC Amphiphiles and the Two Different Types of Implemented Cleavable Linkers

The reported triblock amphiphiles are designed to undergo on-demand splitting exactly at the center of the hydrophilic (A) block, yielding two identical amphiphilic diblock copolymers with the same HLB as the parent TBC. In addition, the TBC amphiphiles contain two enzymatically degradable hydrophobic blocks on both sides of the central hydrophilic block and hence are expected to self-assemble into nanosized flower-like micelles, similar to other triblock amphiphiles.^20−22^ Upon activation by external stimuli and cleavage of the responsive linker at the center of the hydrophilic block, the TBCs will split into two identical DBCs. As these DBC amphiphiles have the same HLB as the original TBC, they should remain assembled as micelles, and only minor cargo release is expected. On the other hand, lowering the molecular weight by half and changing the architecture from triblock to diblock copolymers should significantly increase the unimer–micelle exchange rate.^23−25^ This change in unimer–micelle equilibrium is expected to accelerate enzymatic degradation of the DBCs’ hydrophobic blocks, leading to complete disassembly of the hydrolyzed polymers (Figure 1). To demonstrate the macromolecular transition of the TBC to two DBCs upon splitting, we chose poly(acrylic acid) (PAA) as the hydrophilic polymer and used either UV- or redox-responsive linkers, which were placed at the center of the PAA. These two linkers should allow us to achieve the macromolecular transition by either irradiation of light or in the presence of a reducing agent, respectively. PAA was selected due to its relatively high hydrophilicity, which should facilitate the formation of micelles, rather than hydrogels that are often obtained for other PEG-based triblock systems.^26^ To study the architectural changes and the resulting supramolecular effects with high resolution, we used enzyme-responsive dendrons as the hydrophobic (B) blocks (Figure 1).^27,28^ The dendrons contained hydrophobic ester end-groups (Scheme 1), serving as substrates for a model enzyme—porcine liver esterase (PLE). The ability to control the responsiveness of amphiphiles toward enzymatic degradation by introducing a stimuli-responsive cleavage site within the hydrophilic block opens up the way toward modular design of highly stable and yet enzyme-responsive polymeric nanoassemblies.

## Results and Discussion

The synthesis of the TBCs (Scheme 1A) started by atom transfer radical polymerization (ATRP) of *tert*-butyl acrylate (^t^BA) from a bifunctional initiator that contained the splittable linker at its center.^29−31^ To demonstrate the generality and modularity of our approach, two stimuli-responsive linkers were used: a redox-responsive linker that bears a disulfide bond in its center, which can be cleaved by a reducing agent such as dithiothreitol (DTT), and a UV-responsive linker that contains 4,5-dimethoxy-2-nitrobenzyl (DMNB) linked by an AB_2_ self-immolative spacer.^29,32^ After polymerization, the terminal bromides were substituted into azides, which were conjugated by copper-catalyzed azide–alkyne cycloaddition (CuAAC)^33^ with esterase-cleavable hydrophobic dendrons.^34^ The dendrons were fluorescently labeled with 7-diethylamino-3-carboxy coumarin (7-DEAC) due to its ability to form excimers when the micelles are assembled, as indicated by fluorescence emission maxima at 560 nm (rather than 480 nm, the emission maxima of the free dye).^35^ This red-shifted emission of the fluorescently labeled amphiphiles in the assembled state can supply essential structural information on the micellar mesophase^34−37^ during the transformation from the TBC to DBC amphiphiles. At the last step of the synthesis, the *tert*-butyl protecting groups were removed under acidic conditions, exposing highly hydrophilic carboxylic acids of the poly (acrylic acid) (PAA) backbone (Scheme 1B). In addition to the redox-responsive (SS-TBC) and the UV-responsive (DMNB-TBC) amphiphiles, a nonresponsive TBC with a heptyl chain in its center (C7-TBC) was synthesized and used as a control (Figure S5). All TBCs were obtained in high purity and characterized by NMR, HPLC, SEC, and UV-Vis and fluorescence spectroscopies (see the Supporting Information).

**Figure 2:**
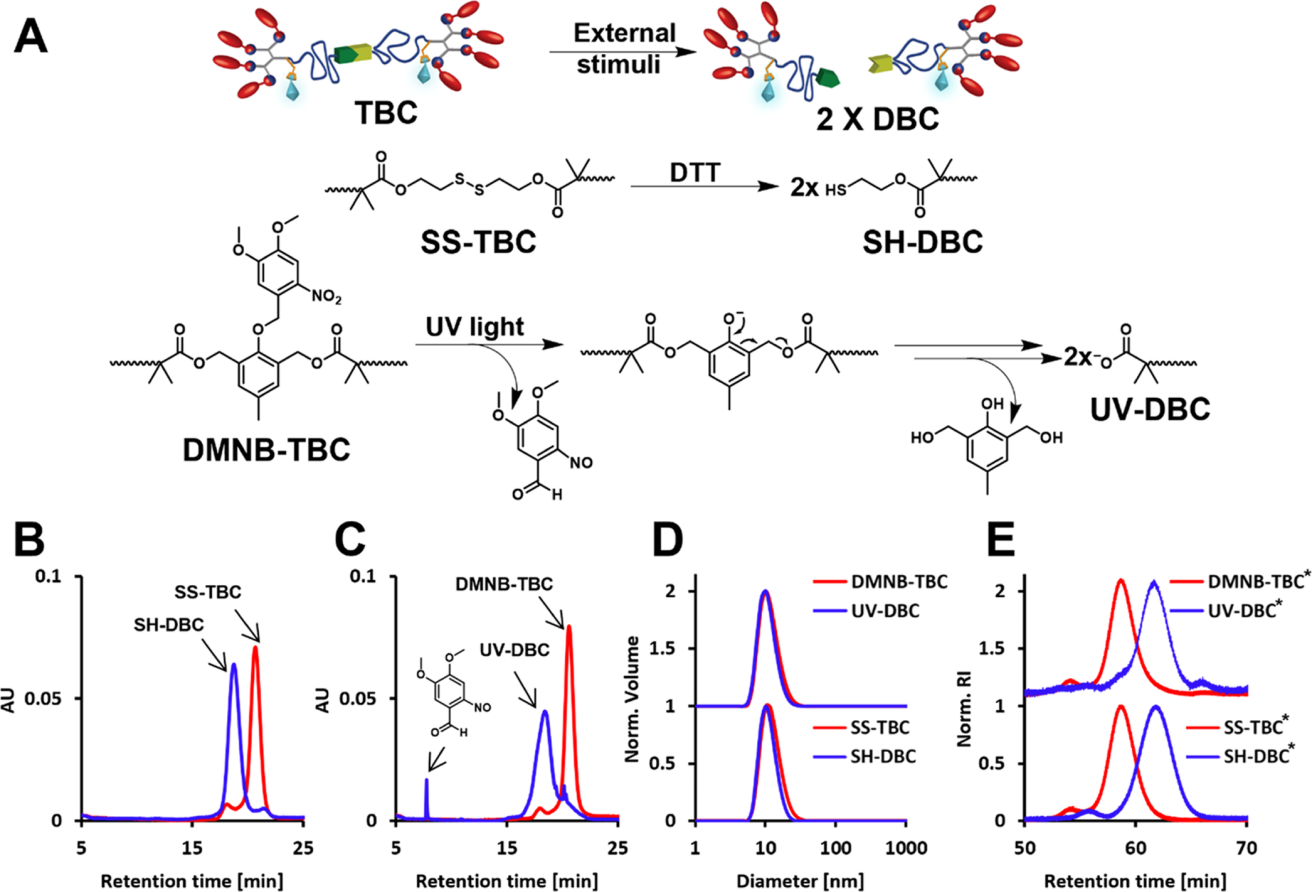
(A) Schematic presentation of stimuli-induced splitting of the hydrophilic blocks in their middle, by DTT or UV light causing the transition from TBC to DBC amphiphiles. Overlays of HPLC chromatograms (taken at 420 nm) of (B) SS-TBC before (red) and after (blue) treatment with DTT and (C) DMNB-TBC before (red) and after (blue) UV irradiation. (D) DLS measurements before (red lines) and after (blue lines) activation of DMNB-TBC (top) and SS-TBC (bottom); ([TBC] = 80 μM, [DTT] = 20 mM). (E) SEC traces of *tert*-butyl-protected DMNB-TBC (DMNB-TBC*, top) and *tert*-butyl-protected SS-TBC (SS-TBC*, bottom) before (red) and after (blue) splitting upon UV irradiation or the addition of DTT (20 mM); ([TBC*] = 10 mg/mL).

With both triblock amphiphiles in hand, we first studied their self-assembly in aqueous media (PBS pH 7.4) by dynamic light scattering (DLS), which showed structures with diameters of 12 ± 4 nm for both SS- and DMNB-TBC-based micelles (Figures 2D and S32). Further validation for the formation of micelles was obtained by transmittance electron microscopy (TEM), which showed spherical structures with similar diameters (Figure S29). Next, we characterized the architectural transition of self-assembled TBCs into DBCs (Figure 2A). First, HPLC analysis confirmed the splitting of the TBCs, showing the disappearance of the starting TBC peaks and the appearance of new peaks with slightly shorter retention times, which were assigned as the DBCs (Figure 2B,2C).

**Figure 3:**
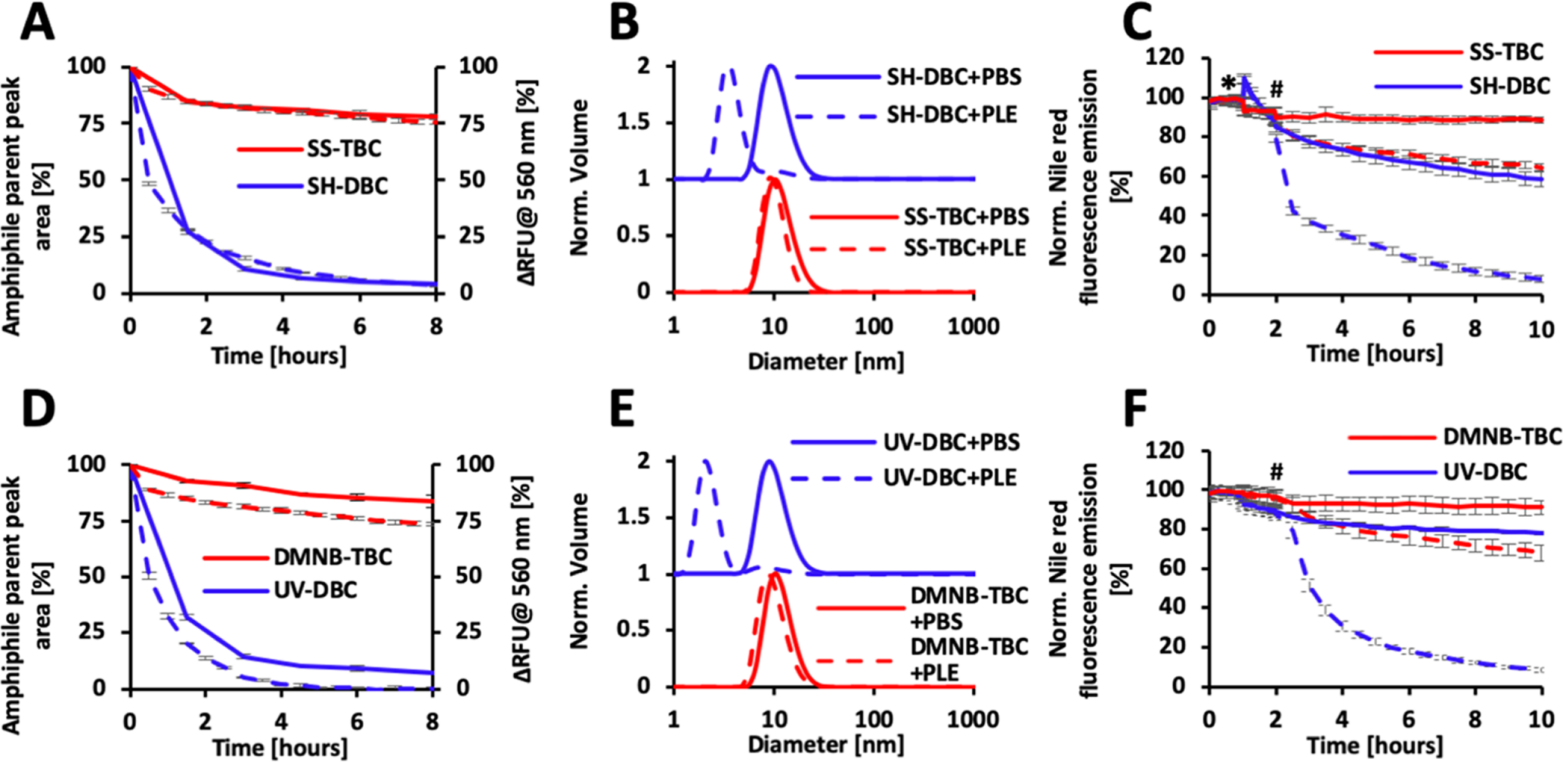
Enzymatic degradation and disassembly of the redox (top row)- and UV (bottom row)-activatable micelles before (red lines) and after (blue lines) splitting. (A, D) Enzymatic degradation profiles as obtained by HPLC (solid lines) and fluorescence spectroscopy (dashed lines). (B, E) DLS measurements after 8 h of incubation with PBS (solid lines) or PLE (dashed lines). (C, F) Nile red release experiments after the addition of PBS (solid lines) or PLE (dashed lines). * Addition of DTT or PBS. ^#^ Addition of PLE or PBS. [TBC] = 80 μM, [PLE] = 0.1 μM, λ_Ex 7-DEAC_ = 420 nm, [Nile red] = 1.5 μM, λ_Ex Nile red_ = 550 nm.

DLS was then used to determine micellar sizes of the DBCs directly after splitting (Figures 2D and S32) and after an additional 8 h of incubation at 37 °C (Figure 3B,E). The transformation to DBC amphiphiles did not lead to a significant change in micellar structures, as nearly identical diameters of 11 ± 3 nm were observed for both SH- and UV-DBC-based micelles. The critical micelle concentrations (CMCs) were then determined using the Nile red method^38^ and were found to be around 3 μM for the two TBCs, while SH- and UV-DBC amphiphiles showed a slight increase to around 4–5 μM. This points out that the architectural transition from TBC to DBC is not accompanied by a significant change in the thermodynamic stability of the micelles.

SEC was then used to verify the splitting of the amphiphiles. Due to the strong column interaction of the multiple carboxylic acids of PAA, SEC was performed for the *tert*-butyl-protected amphiphiles. SEC chromatograms confirmed the ability of the stimuli-responsive linkers to be cleaved, showing the disappearance of the TBCs and the appearance of DBCs with half the molecular weight (Figures 2E, S23, and S24).

Once the architectural transition from TBC to DBC was confirmed, we tested its effect on the enzymatic degradation of the amphiphiles. Based on the inspiring work of Lodge and Bates, which studied the effects of molecular weight and architecture on unimer–micelle exchange rates,^25^ we expected the change from TBC to DBC amphiphiles to lead to much faster exchange rates. As the enzymatic degradation process strongly depends on the ability of the enzyme to reach its hydrophobic substrates, this change in architecture and the resulting increase in exchange rates should significantly accelerate the enzymatic degradation.^16,18^ The enzymatic hydrolysis of the hydrophobic end-groups of the dendron by the activating enzyme PLE should result in a significant decrease in the overall hydrophobicity of the amphiphiles, leading to the complete disassembly of the micelles.

Micellar solutions of both TBCs and DBCs were treated with PLE and incubated at 37 °C for 8 h. Using HPLC, we could directly quantify the enzymatic degradation by monitoring the peak areas of the amphiphiles at 420 nm, which is the absorbance wavelength of the labeling dye 7-DEAC. While SH-DBC amphiphiles were fully degraded within less than 4 h (Figure 3A, solid lines), SS-TBC showed only a limited degree of degradation over 8 h (∼25%). In parallel, we measured the change in 7-DEAC fluorescence emission intensity at 560 nm, which is a characteristic of the self-assembled state. Upon disassembly of the micelles, the diffusion of the degraded hydrophilic polymers away from each other results in the decrease in excimer intensity (Figure 3A, dashed lines). The fluorescence measurements correlate very well with the kinetic data obtained by HPLC, showing that indeed, the enzymatic degradation led to micellar disassembly. DLS measurements further confirmed the disassembly of the SH-DBC micelles, while the SS-TBC micelles remained intact in the presence of PLE (Figure 3B). The photoresponsive DMNB-TBC and its corresponding DBC showed very similar trends (Figure 3D,3E). Importantly, when the control amphiphile C7-TBC, which lacks the cleavable linker, was incubated with PLE alone or with PLE in the presence of DTT or after UV irradiation, only minor differences in the enzymatic degradation rates and size were observed (Figures S30 and S33). In addition, no hydrolysis was observed for all three TBC and related DBC amphiphiles in the absence of the activating enzyme (Figure S31). These control experiments demonstrate that the acceleration of the enzymatic degradation rates was solely due to the splitting of the TBC amphiphiles and not because of the background effects of the applied stimuli.

In light of the potential application of this architectural transition as a release mechanism for controlled drug delivery systems, we wanted to test if the transition from TBC to DBC will affect the release of the encapsulated hydrophobic cargo. Nile red was selected as a model cargo, as it is highly emissive in nonpolar hydrophobic microenvironments such as a micelle and has a very weak emission in polar microenvironments such as PBS. Nile red was encapsulated within the TBC micelles and as expected showed high fluorescence intensity. The TBC micelles were then incubated with DTT or UV-irradiated to induce the splitting into DBCs, followed by addition of PLE. While only minor changes in fluorescence were observed for both types of TBC-based micelles in the presence of the activating enzyme, significant decreases in fluorescence emissions, indicating the release of Nile red, were observed for the DBC-based micelles (Figure 3C,F). These results demonstrate that the splitting of the TBCs into DBCs can be used to control the enzymatic disassembly rates of the micelles and the induced cargo release.

**Figure 4:**
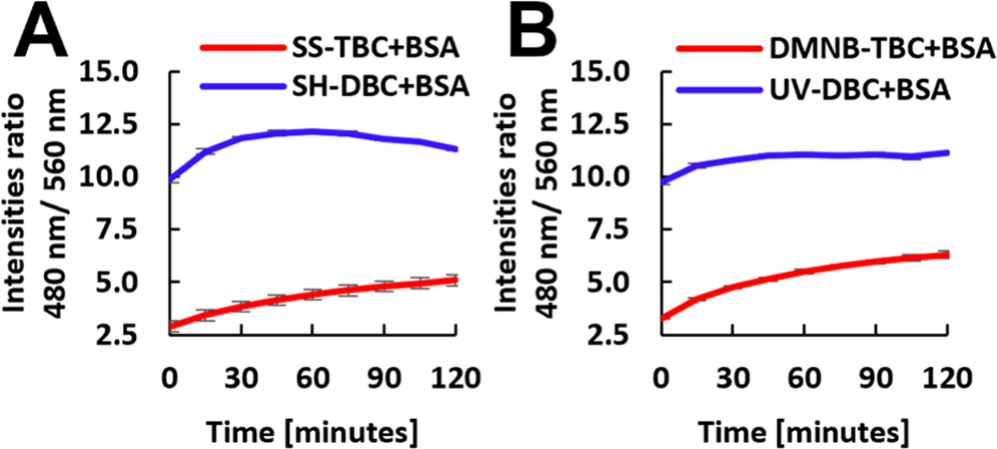
Micelle destabilization by BSA. Unimer/micelle fluorescence intensity ratio (480 nm/560 nm) over time upon the addition of BSA into micellar solutions of (A) SS-TBC (red) and SH-DBC (blue) and (B) DMNB-TBC (red) and UV-DBC (blue). [TBC] = 80 μM, [BSA] = 5.5 mg/mL, λ_Ex_ = 420 nm.

After confirming the ability of the architectural transition from TBC to DBC to affect the interaction of the hydrophobic blocks with PLE, we wished to examine the generality of this molecular strategy to tune the interactions with other proteins. Bovine serum albumin (BSA), a transport protein that is known to interact with hydrophobic moieties,^39,40^ was selected as a model protein, as it was shown to interact with the hydrophobic domains of our amphiphiles.^41^ The self-reporting spectral mechanism of the micelles was utilized to evaluate the differences in the degree of interactions of micelles with BSA before and after splitting. Micellar solutions of TBCs and DBCs were treated with either BSA (Figure 4) or PBS (Figure S34), and fluorescence spectra were recorded every 15 minutes for 2 h. To qualitatively analyze the degree of micellar destabilization, we calculated the ratio between fluorescence intensities at 480 and 560 nm, which correspond to 7-DEAC emissions, while the amphiphiles are in their unimer and micellar states, respectively. Interactions of BSA with the hydrophobic blocks of the amphiphiles should tilt the unimer–micelle equilibrium toward the unimer state and in addition cause a significant increase in the fluorescence of the 7-DEAC dye due to a solvatochromic effect, causing an overall increase in the unimer/micelle emission ratio.^42^ Based on the fluorescence spectra showing that the unimer/micelle fluorescence ratio for the TBC-based micelles is roughly half of the ratio for the DBC ones, it is clear that DBC micelles were more sensitive toward BSA in comparison with TBC micelles. The comparable trends of the increased degree of interactions for both PLE and BSA with DBC micelles indicate that these protein–amphiphile interactions follow a similar mechanism. As proteins cannot penetrate through the hydrophilic shell, unimers are required to escape the micelles to interact with them.^16,43^ The observed dependence of polymer–protein interactions on the polymeric architecture opens up the way for using architectural change as a modular tool for controlling the protein responsiveness of the hydrophobic blocks.

## Conclusions

The need to balance between stability and enzymatic degradability of polymeric micelles has been a key obstacle toward their translation into nanocarriers that can be activated by disease-associated enzymes. To address this challenge, we developed a modular approach based on stimuli-induced splitting of amphiphilic triblock copolymers into two equivalent diblock amphiphiles. This architectural transition affects the kinetic stability of the assemblies, as the faster unimer–micelle exchange rates of the smaller diblock amphiphiles make their hydrophobic blocks more accessible to proteins. As a proof of concept, we designed fluorescently labeled TBC amphiphiles with enzymatically degradable dendritic end-groups and a single cleavable linker, which was located exactly in the middle of the hydrophilic block. The high molecular precision that emerges from using dendrons as the hydrophobic blocks, together with the self-reporting spectral mechanism, allowed us to carefully study the transition from TBC- to DBC-based micelles in response to interactions with two proteins: an enzyme (PLE) and a transport protein (BSA). We show that this induced architectural change is not affecting the HLB of the split amphiphiles, the size of the micelles, or their thermodynamic properties. However, the stimuli-induced architectural transition from TBC to two DBC amphiphiles drastically decreases the kinetic stability of their micelles toward interacting with enzymes. The ability to significantly affect the enzymatic responsiveness is demonstrated by the substantially faster enzymatic degradation and micellar disassembly of the DBC amphiphiles in comparison with the TBC based micelles. To show the generality of our approach, we designed and studied TBC amphiphiles that can respond to two different types of stimuli—reducing agent and UV light. Our results show that this approach can potentially allow simple tailoring of TBC amphiphiles to different types of stimuli and enzymes by applying the appropriate splittable linkers and end-groups. The triblock to diblock architectural transition opens up the way for the design of extremely stable and yet highly responsive polymeric assemblies for various applications ranging from biomedicine to agriculture, where controlling the interaction between polymers and proteins is essential.

## Supporting Information Available

Synthetic procedures, amphiphile and micelle characterization data, detailed experimental protocols, and control experiments (PDF)

## Abbreviations Used

CMC: critical micelle concentration
DLS: dynamic light scattering
HPLC: high-pressure liquid chromatography
