# Supplementary material for: Stimuli-Induced Architectural Transition as a Tool for Controlling the Enzymatic Degradability of Polymeric Micelles
Source: ACS Polym Au. 2022 Jul 27;2(5):380–6. doi: 10.1021/acspolymersau.2c00023 (PMC9955281; doi:10.1021/acspolymersau.2c00023)
Supplement: Supplementary file 1 — lg2c00023_si_001.pdf [file lg2c00023_si_001.pdf]

Stimuli-induced architectural transition as a tool for controlling the enzymatic degradability of polymeric micelles

Gadi Slor‡[1,2], Shahar Tevet‡[1,2,3,4] and Roey J. Amir*[1,2,3,4]

1 Department of Organic Chemistry, School of Chemistry, Faculty of Exact Sciences, Tel-Aviv University, Tel-Aviv 6997801, Israel

2 Tel-Aviv University Center for Nanoscience and Nanotechnology, Tel-Aviv University, Tel-Aviv 6997801, Israel

3 ADAMA Center for Novel Delivery Systems in Crop Protection, Tel-Aviv University, Tel-Aviv 6997801, Israel

4 The Center for Physics and Chemistry of Living Systems, Tel-Aviv University, Tel-Aviv 6997801, Israel

Supplementary Information

Table of Contents

Instrumentation and Materials **2**

Instrumentation *2*

Materials *2*

Synthesis **3**

Characterization of Polymer-dendron amphiphiles **24**

HPLC measurements *24*

Size exclusion chromatography (SEC) *26*

Splitting of TBC amphiphiles **29**

Critical micelles' concentration (CMC) **30**

Enzymatic degradation experiments **33**

Dynamic light scattering **35**

Nile red release experiments **36**

Micellar stability in the presence of BSA **36**

References **37**

Instrumentation and Materials

Instrumentation

HPLC: All measurements were recorded on a Waters Alliance e2695 separations module equipped with a Waters 2998 photodiode array detector. All solvents were purchased from Bio-Lab Chemicals and were used as received. All solvents are HPLC grade. **^{1}H and ^{13}C NMR:** spectra were recorded on Bruker Avance I and Avance III 400MHz (and 100MHz) spectrometers as indicated. Chemical shifts are reported in ppm and referenced to the solvent. **FTIR:** IR spectra were record on a FTIR ATR spectrometer – TENSOR 27 by Bruker. **TEM:** Images were taken by a JEM-1400Plus TEM at 120kV. **SEC:** All measurements were recorded on Viscotek GPCmax by Malvern using refractive index detector and PEG standards (purchased from Sigma-Aldrich) were used for calibration. **Fluorescence spectra:** CMC measurements and micellar stability in the presence of BSA experiments were recorded on a TECAN Infinite M200Pro device, enzymatic degradation experiments and Nile red release experiments were recorded on an Agilent Technologies Cary Eclipse Fluorescence Spectrometer using quartz cuvettes. **DLS:** All measurements were recorded on a Malvern Zetasizer NanoZS.

Materials

3-mercaptopropionic acid (98%), 2,2-dimethoxy-2-phenylacetophenone (DMPA, 99%), 4-(Dimethylamino)pyridine (DMAP, 99%), Fmoc-L-Lys(Boc)-OH (98%), N-Hydroxysuccinimide (NHS, 99%), Copper(I) bromide (CuBr, 98%), N,N,N′,N′′,N′′-Pentamethyldiethylenetriamine (PMDETA, 99%), Bis[2-(2′-bromoisobutyryloxy)ethyl]disulfide, α-Bromoisobutyryl bromide, 4,5-Dimethoxy-2-nitrobenzyl bromide, 2,6-Bis(hydroxymethyl)-p-cresol, Bovine Serum Albumin (BSA), Porcine liver esterase (PLE) and SephadexR LH20 were purchased from Sigma-Aldrich. 1-hexanol (98%) was purchased from Acros Organics. Propargyl bromide (80% in toluene), chlorotriphenylmethane (Trt-Cl, 98%), 4-nitrophenol (99%), triethylsilane (98%), N,N'-dicyclohexylcarbodiimide (DCC, 99%), propargyl amine (98%), tert-Butyl acrylate (tBA, 99%), triethylamine and anhydrous K_2CO_3 (99%) were purchased from Alfa Aesar. 3,5-dihydroxy benzoic acid was purchased from Apollo scientific. Cystamine hydrochloride (98%), Potassium hydroxide, Diisopropylethylamine (DIPEA) and sodium azide (NaN_3) were purchased from Merck. Silica Gel 60A, 0.040-0.063mm, sodium hydroxide, Anhydrous Na_2SO_4 (granular, 10-60mesh), piperidine (peptide synthesis), N,N-Dimethylformamide (DMF, peptide synthesis), Trifluoroacetic acid (TFA, HPLC grade) and all solvents were purchased from Bio-Lab and were used as received. Deuterated solvents for NMR were purchased from Cambridge Isotope Laboratories (CIL), Inc.

Synthesis

ATRP initiators:

Redox-responsive initiator was purchased from Sigma-Aldrich and was used as received.

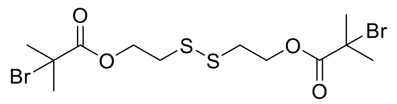

Figure S1: Redox-responsive initiator.

Photo-responsive initiator was synthesized as previously reported[1] and the spectroscopic characterization correlated well with these reports. ^{1}H NMR (400 MHz, Chloroform-*d*): δ 7.77 (s, 1H, Ar-***H***), 7.62 (s, 1H, Ar-***H***), 7.30 (s, 2H, Ar-***H***), 5.39 (s, 2H, Ar-C$\boldsymbol{H}_2$-O-), 5.24 (s, 4H, Ar-C$\boldsymbol{H}_2$-O-), 4.06 (s, 3H, -O-C$\boldsymbol{H}_3$), 3.98 (s, 3H, -O-C$\boldsymbol{H}_3$), 2.37 (s, 3H, Ar-C$\boldsymbol{H}_3$), 1.89 (s, 12H, -CO-C-(C$\boldsymbol{H}_3$)$_2$-Br). ^{13}C NMR (DMSO-d6, 100 MHz): δ 153.6, 151.2, 147.5, 138.6, 134.7, 132.9, 129.2, 128.1, 109.8, 107.9, 72.2, 58.2, 56.3, 56.1, 20.7. MS (m/z, [M+Na]$^+$, ESI): calculated mass (+Na): 386.1216, found: 386.1218.

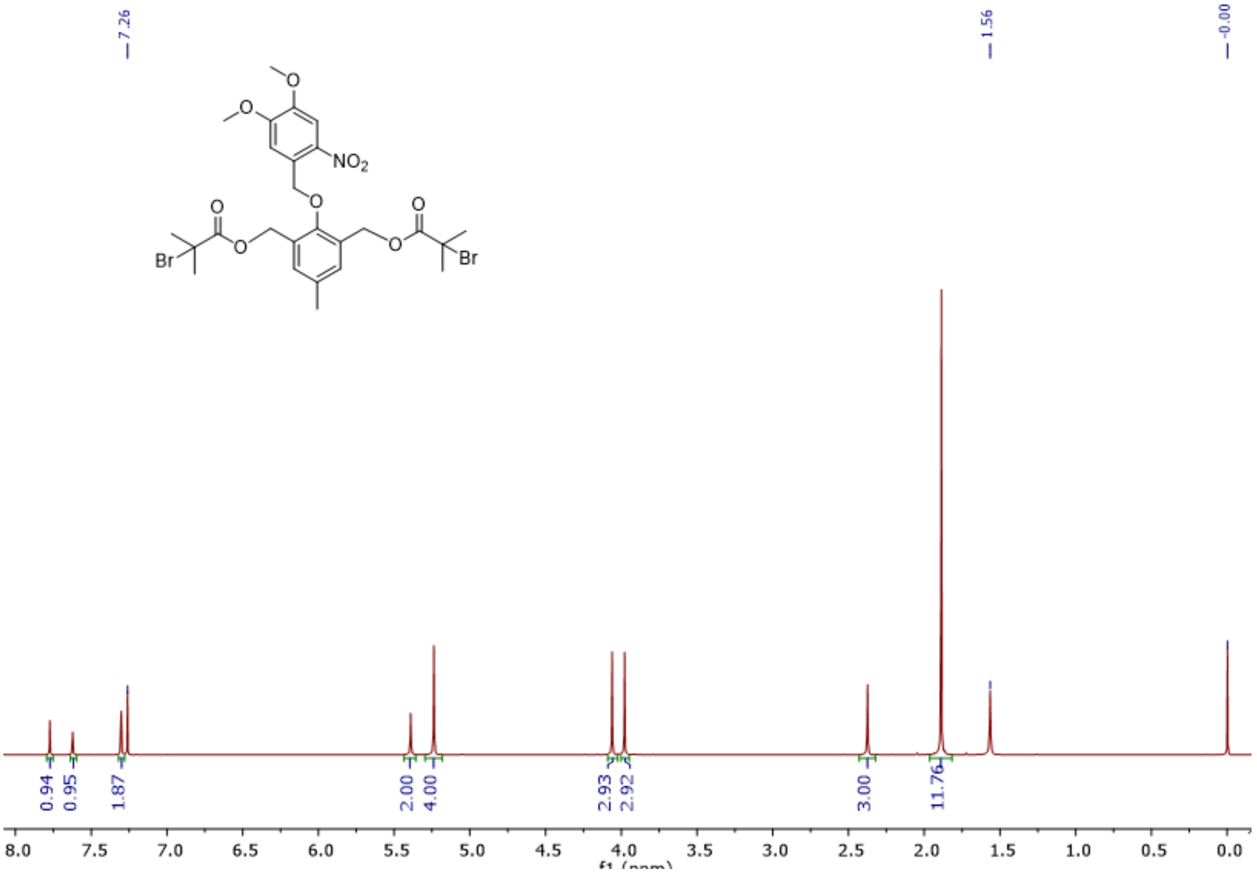

Figure S2: ^{1}H-NMR spectra of photo-responsive initiator.

C7 non-responsive initiator

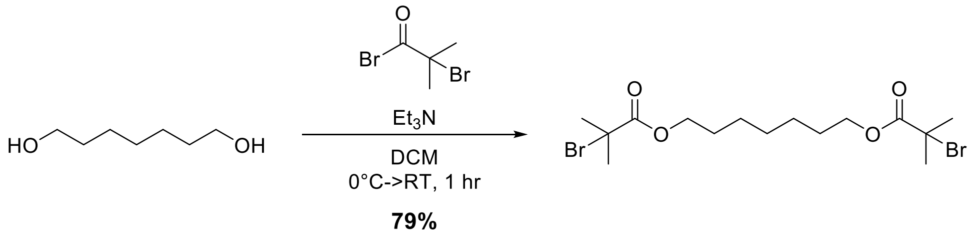

Figure S3: Synthetic scheme for the preparation of C7 non-responsive initiator.

1,7-heptanediol (0.7 mL, 5.03 mmol) and Et_3N (2.1 mL, 15.10 mmol) were dissolved in DCM (30ml) and cooled to 0°C. α-Bromoisobutyryl bromide (1.9 mL, 15.10 mmol) was dissolved in DCM (10ml) and added dropwise. Reaction was allowed to heat to room temperature and stirred for 1hr. Organic phase was washed with saturated NH_4Cl solution and saturated $NaHCO_3$ solution (20 mL each), dried over Na_2SO_4 and solvents were removed under reduced pressure. Product was purified by silica column (90:10 Hex:EA, TLC plates were stained with $KMnO_4$). Product was obtained as colorless oil in 79% yield (1.7 gr).

^{1}H NMR (400 MHz, Chloroform-*d*) δ 4.15 (t, *J* = 6.6 Hz, 4H, CH_2-C***H***$_2$-O-), 1.91 (s, 12H, -CO-C-(C***H***$_3$)$_2$-Br), 1.66 (m, 4H, CH_2-C***H***$_2$- CH_2-O-), 1.46 – 1.28 (m, 6H, O-CH_2- CH_2- (C***H***$_2$)$_3$-CH_2-CH_2-O).^{13}C NMR (100 MHz, Chloroform-*d*) δ 171.7, 65.9, 55.9, 30.7, 28.6, 28.2, 25.6. MS (m/z, [M+Na]$^+$, ESI): calculated: 453.0, found: 453.1.

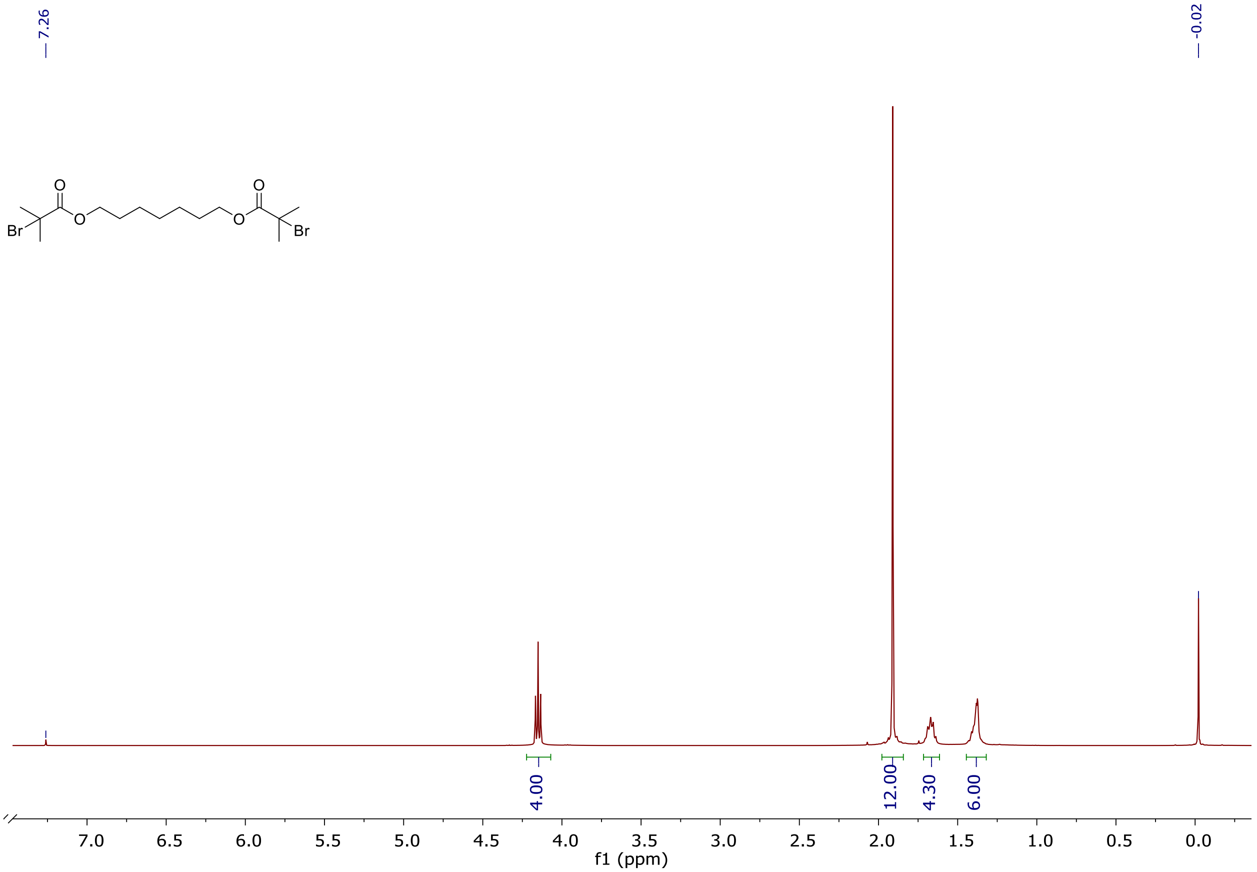

Figure S4: ^{1}H-NMR spectra of C7 non-responsive initiator.

Preparation of azide functionalized PtBAs:

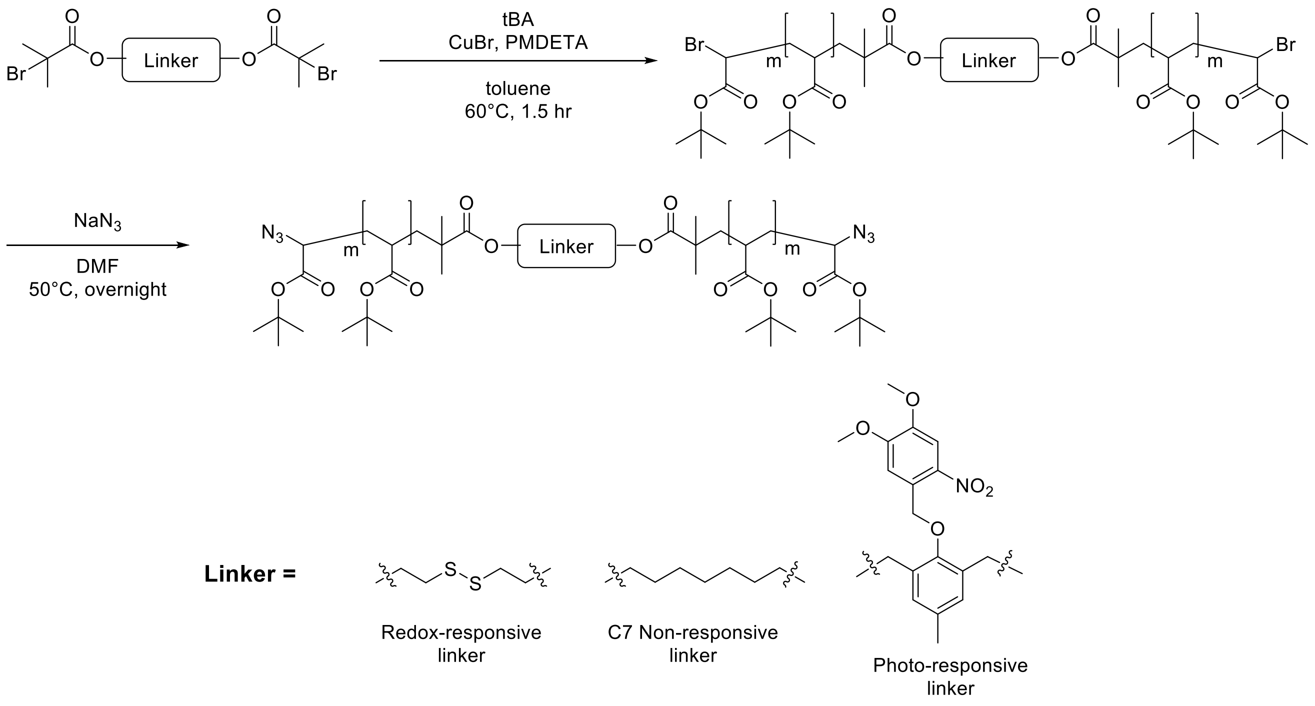

Figure S5: Synthetic scheme for the preparation of di-azide functionalized PtBA.

General procedure for ATRP polymerization:

50ml tert butyl acrylate (tBA) were washed with with 5% NaOH aqueous solution (3x40 mL) and then with water (40 mL), dried over $MgSO_4$ and distilled in vacuum (~60°C, 30 mbar).

The desired initiator (1 eq) was dissolved in 2 ml toluene and in separate vial PMDETA (2 eq) was dissolved in tBA (50 eq) and 0.5 ml toluene. Both vials were purged with nitrogen for 15 minutes. CuBr (2 eq) was loaded in round buttom shlenk flask, which was evacuated and backfilled with nitrogen three times and left under nitrogen atmosphere. PMDETA and tBA mixture were added using nitrogen flushed syringe and needle and stirred with CuBr for 20 minutes at room temperature until solution became clear and greenish. The initiator solution was then added using nitrogen flushed syringe and needle and the flask was submerged in an oil bath preheated to 60°C and stirred for 1.5 hr. Reaction was stopped by opening the flask to air and cooling it in an ice bath. Reaction mixture was then filtered through Celite and neutral alumina, concentrated in vacuum, redissolved in 50 mL THF and precipitated into 500 mL ice cold mixture of water and MeOH (1:1 v/v). Solvents were decanted and another precipitation was done. After decanting the water: MeOH mixture the

white residue was dissolved in DCM, dried over Na_2SO_4 and evaporated to dryness. The polymers were obtained as white solids. The degree of polymerization (DP) was determined using ^1H NMR spectroscopy by comparing the integration of the four methyl groups of the initiator (~1.1 ppm, calibrated as 12H) to the methine (CH) of the polymer backbone (~2.0-2.5 ppm).

General procedure for azidation of PtBA:

Bromide functionalized PtBA (1 eq) was dissolved in DMF (10-15 ml per 1 gr polymer), NaN_3 (20 eq) was added and reaction was stirred overnight at 50°C. Reaction was then cooled to room temperature and diluted with ether (150 mL) which was washed with water (3x100 mL), dried over Na_2SO_4 and evaporated to dryness. The products were obtained as white solids.

SS-PtBA-Br:

Redox-responsive initiator (502 mg, 1.111 mmol), PMDETA (470 µL, 2.22 mmol), tBA (8.13 mL, 55.5 mmol) and CuBr (318 mg, 2.22 mmol) were reacted according to the general procedure. The polymer was obtained as white solid in 79% yield (5.5 gr).

^1H NMR (400 MHz, Chloroform-*d*) δ 4.30 (br s, 4H, CH_2-C$\boldsymbol{H_2}$-O), 4.10 (m, 2H, -C***H***-Br), 3.03 – 2.80 (t, J = 8.0 Hz 4H, CH_2-C$\boldsymbol{H_2}$-S-), 2.44-2.06 (brs, 43H, PtBA backbone -C***H***-CO), 2.02-1.17 (m, 503H, PtBA backbone –C$\boldsymbol{H_2}$-CH- + -O-C(C$\boldsymbol{H}_3$)$_3$), 1.13 (s, 12H, -CO-C-(C$\boldsymbol{H}_3$)$_2$-PtBA). DP= 45, Monomer conversion = 90%, SEC (DMF + 25mM NH_4Ac): Mn = 3.6 kDa, Đ = 1.12, Expected Mn = 6.3 kDa.

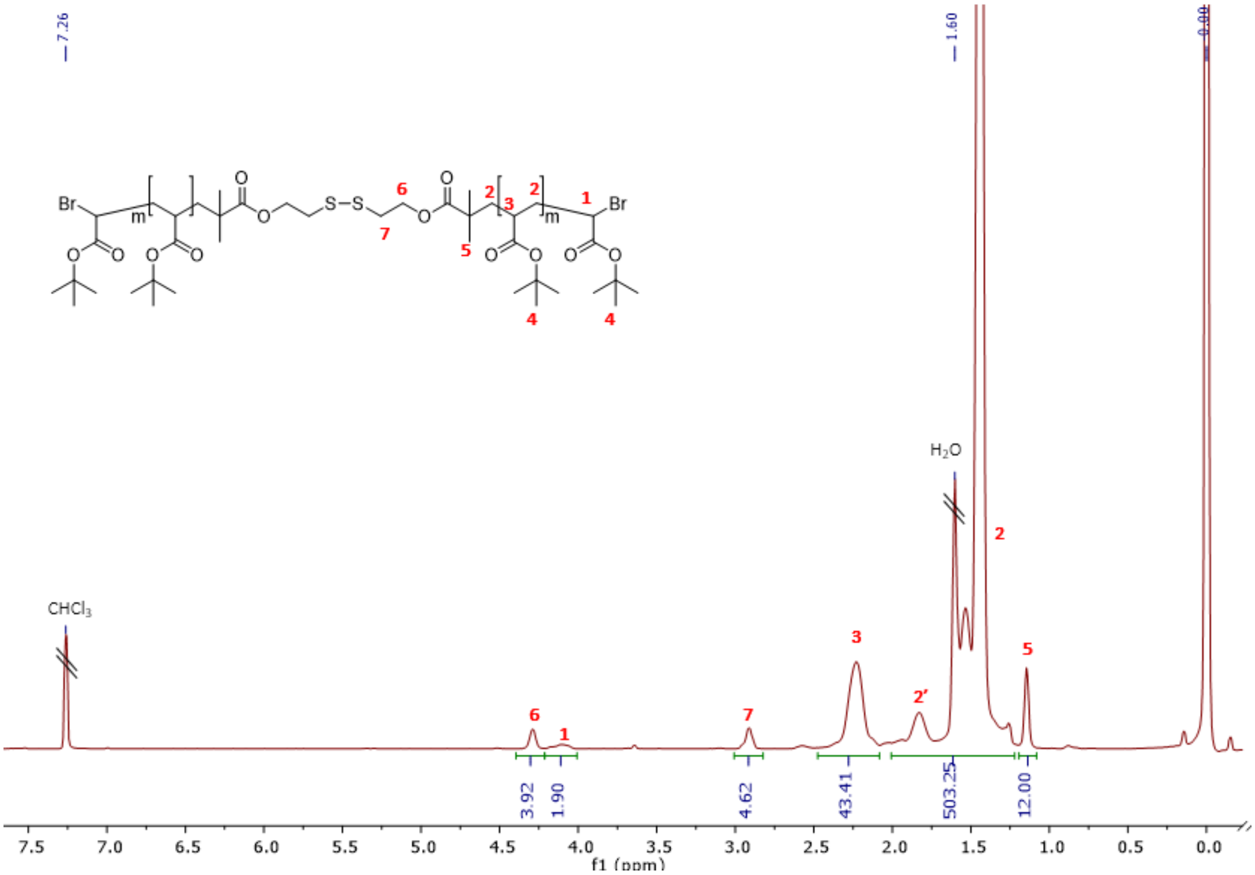

Figure S6: ¹H-NMR spectra of SS-PtBA-Br.

DMNB-PtBA-Br:

Photo-responsive initiator (200mg, 0.30mmol), PMDETA (128µL, 0.60mmol), tBA (2.21mL, 15.12mmol) and CuBr (87mg, 0.60mmol) were reacted according to the general procedure. The polymer was obtained as white solid in 73% yield (1.4 gr).

^{1}H NMR (400 MHz, Chloroform-*d*) δ 7.75 (s, 1H, Ar-***H***), 7.61 (s, 1H, Ar-***H***), 7.24 (s, 2H, Ar-***H***), 5.29 (s, 2H, Ar-C$\boldsymbol{H_2}$-O-), 5.09 (s, 4H, Ar-C$\boldsymbol{H_2}$-O-), 4.20 – 4.04 (m, 2H, -C***H***-Br), 4.03 (s, 3H, -O-C$\boldsymbol{H_3}$), 3.96 (s, 3H, -O-C$\boldsymbol{H_3}$),2.34 (s, 3H, Ar-C$\boldsymbol{H_3}$), 2.31-2.10 (brs, 41H, PtBA backbone -C***H***-CO), 1.93-1.17 (m, 463H, PtBA backbone –C$\boldsymbol{H_2}$-CH- + -O-C(C$\boldsymbol{H_3}$)$_3$), 1.08 (s, 12H, -CO-C-(C$\boldsymbol{H_3}$)$_2$-PtBA). DP = 44, Monomer conversion = 88%, SEC (DMF + 25mM NH_4Ac): Mn = 3.6 kDa, Đ = 1.11, Expected Mn = 6.4 kDa.

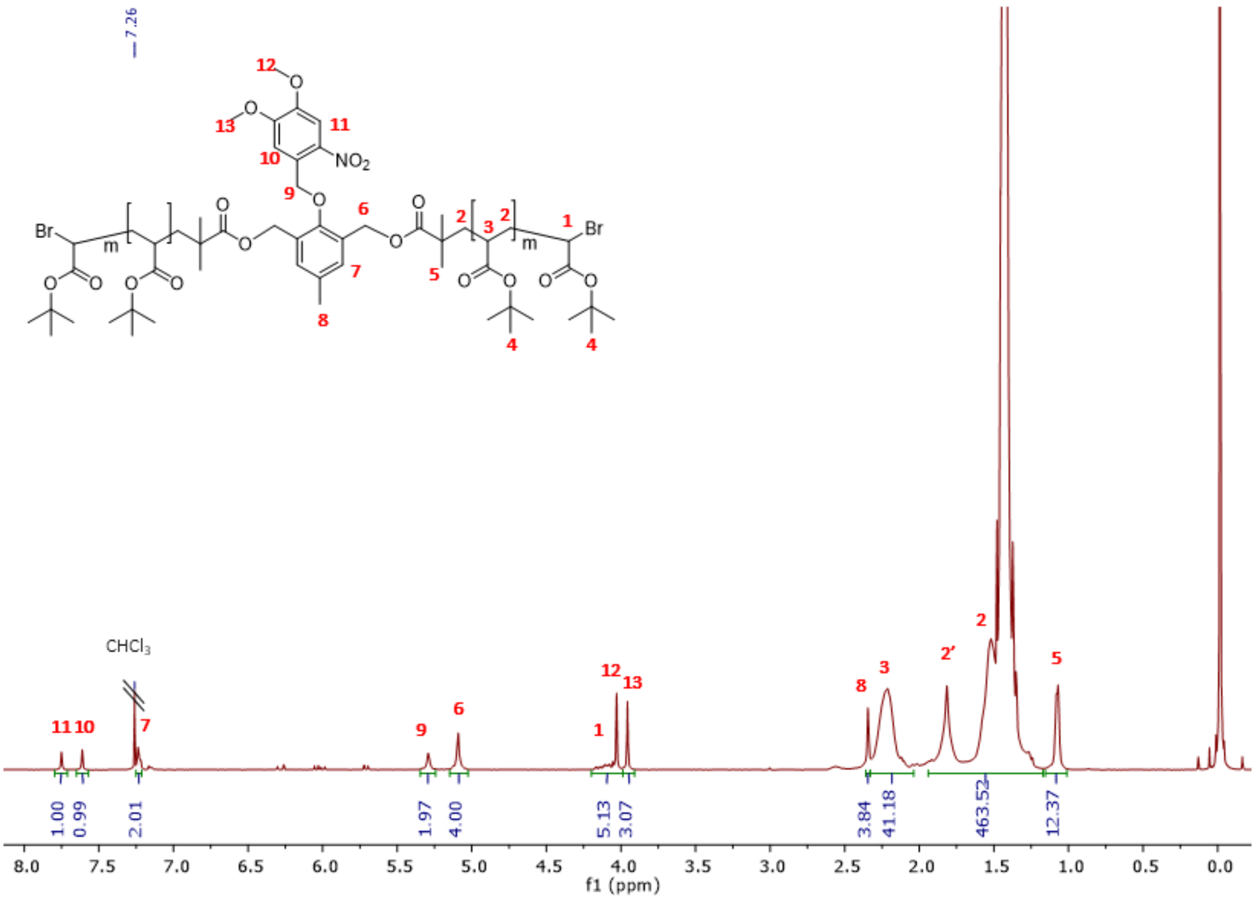

Figure S7: [1]*H-NMR spectra of DMNB-PtBA-Br.*

C7-PtBA-Br:

C7 non-responsive initiator (275mg, 0.61mmol), PMDETA (271µL, 1.22mmol), tBA (4.70 mL, 30.5 mmol) and CuBr (184 mg, 1.22 mmol) were reacted according to the general procedure. The polymer was obtained as white solid in 77% yield (3.3 gr).

^{1}H NMR (400 MHz, Chloroform-*d*) δ 4.29 – 3.88 (m, 6H, CH_2-C$\boldsymbol{H_2}$-O-+ -C$\boldsymbol{H}$-Br), 2.54 – 2.05 (brs, 49H, PtBA backbone -C$\boldsymbol{H}$-CO), 2.03-1.18 (m, 522H, O-CH_2-(C$\boldsymbol{H_2}$)$_5$-CH_2-O + PtBA backbone –C$\boldsymbol{H_2}$-CH- + -O-C(C$\boldsymbol{H_3}$)$_3$), 1.12 (s, 12H, -CO-C-(C$\boldsymbol{H_3}$)$_2$-PtBA). DP = 50, Monomer conversion = 100%, SEC (DMF + 25mM NH_4Ac): Mn = 4.7 kDa, Đ = 1.14, Expected Mn = 7.0 kDa.

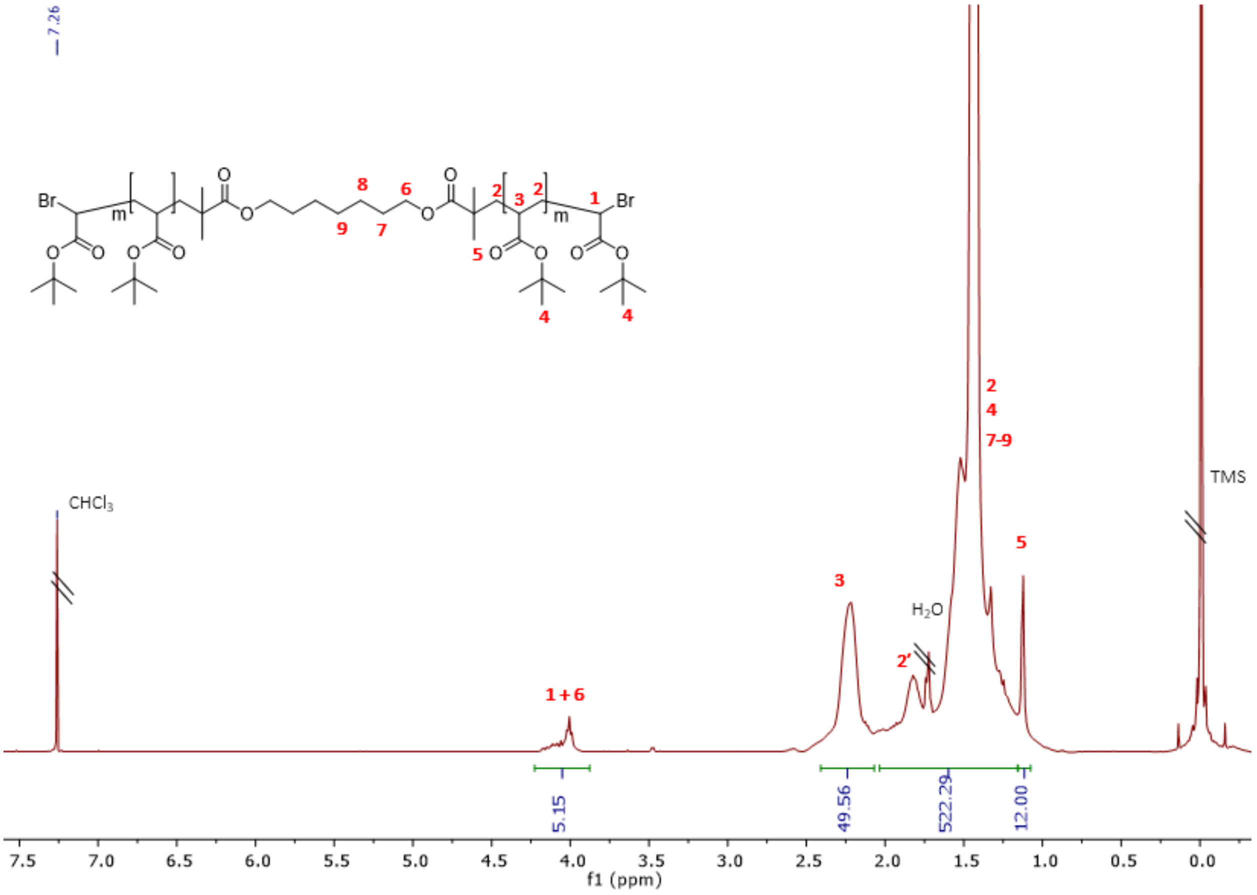

<u>Figure S8</u>: ^{1}H-NMR spectra of C7-PtBA-Br.

SS-PtBA-N_3:

SS-PtBA-Br (1.1 gr, 0.170 mmol) and NaN_3 (224 mg, 3.40 mmol) were reacted according to the general procedure. The product was obtained as white solid in 94% yield (1.03 gr).

^{1}H NMR (400 MHz, Chloroform-*d*) δ 4.29 (br s, 4H, CH_2-C***H₂***-O), 3.83 – 3.57 (m, 2H, -C***H***-N_3), 2.91 (br s, 4H, CH_2-C***H₂***-S-), 2.40-2.12 (brs, 38H, PtBA backbone -C***H***-CO), 2.03-1.21 (m, 486H, PtBA backbone –C***H₂***-CH- + -O-C(C***H₃***)$_3$), 1.14 (s, 12H, -CO-C-(C***H₃***)$_2$-PtBA). SEC (DMF + 25mM NH_4Ac): Mn = 3.7 kDa, Đ = 1.12, Expected Mn = 6.3 kDa.

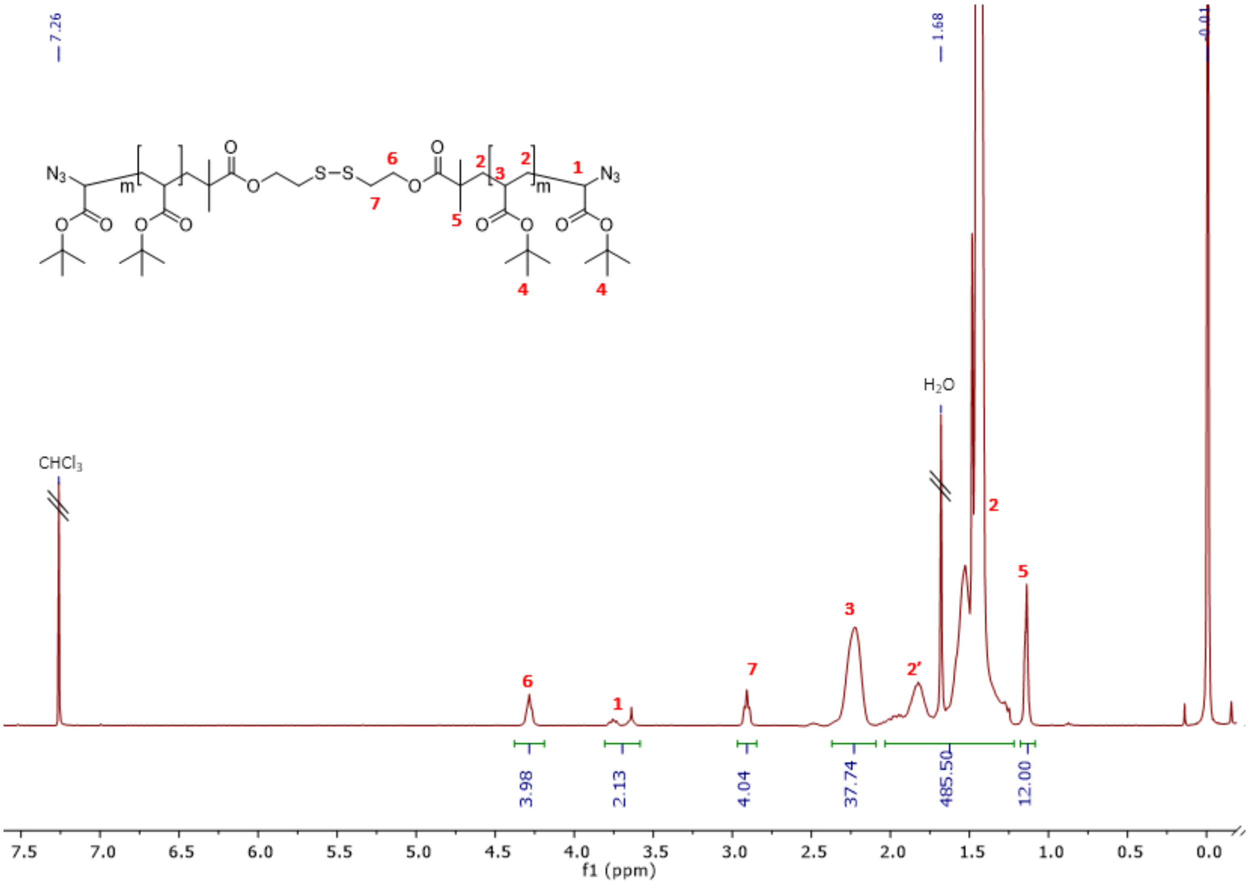

Figure S9: ^{1}H-NMR spectra of SS-PtBA-N_3.

DMNB-PtBA-N_3:

DMNB-PtBA-Br (1.1gr, 0.172mmol) and NaN_3 (224mg, 3.44mmol) were reacted according to the general procedure. The product was obtained as white solid in 91% yield (1.0 gr).

^{1}H NMR (400 MHz, Chloroform-*d*) δ 7.75 (s, 1H, Ar-***H***), 7.61 (s, 1H, Ar-***H***), 7.23 (s, 2H, Ar-***H***), 5.29 (s, 2H, Ar-C***H₂***-O-), 5.09 (s, 4H, Ar-C***H₂***-O-), 4.03 (s, 3H, -O-C$\boldsymbol{H}_3$), 3.96 (s, 3H, -O-C$\boldsymbol{H}_3$), 3.83 – 3.55 (m, 2H, -C***H***-N_3), 2.35 (s, 3H, Ar-C$\boldsymbol{H}_3$), 2.30-2.09 (brs, 40H, PtBA backbone -C***H***-CO), 2.01 – 1.20 (m, 460H, PtBA backbone –C$\boldsymbol{H}_2$-CH- + -O-C(C$\boldsymbol{H}_3$)$_3$), 1.08 (s, 12H, -CO-C-(C$\boldsymbol{H}_3$)$_2$-PtBA). SEC (DMF + 25mM NH_4Ac): Mn = 3.7 kDa, Đ = 1.14, Expected Mn = 6.4 kDa.

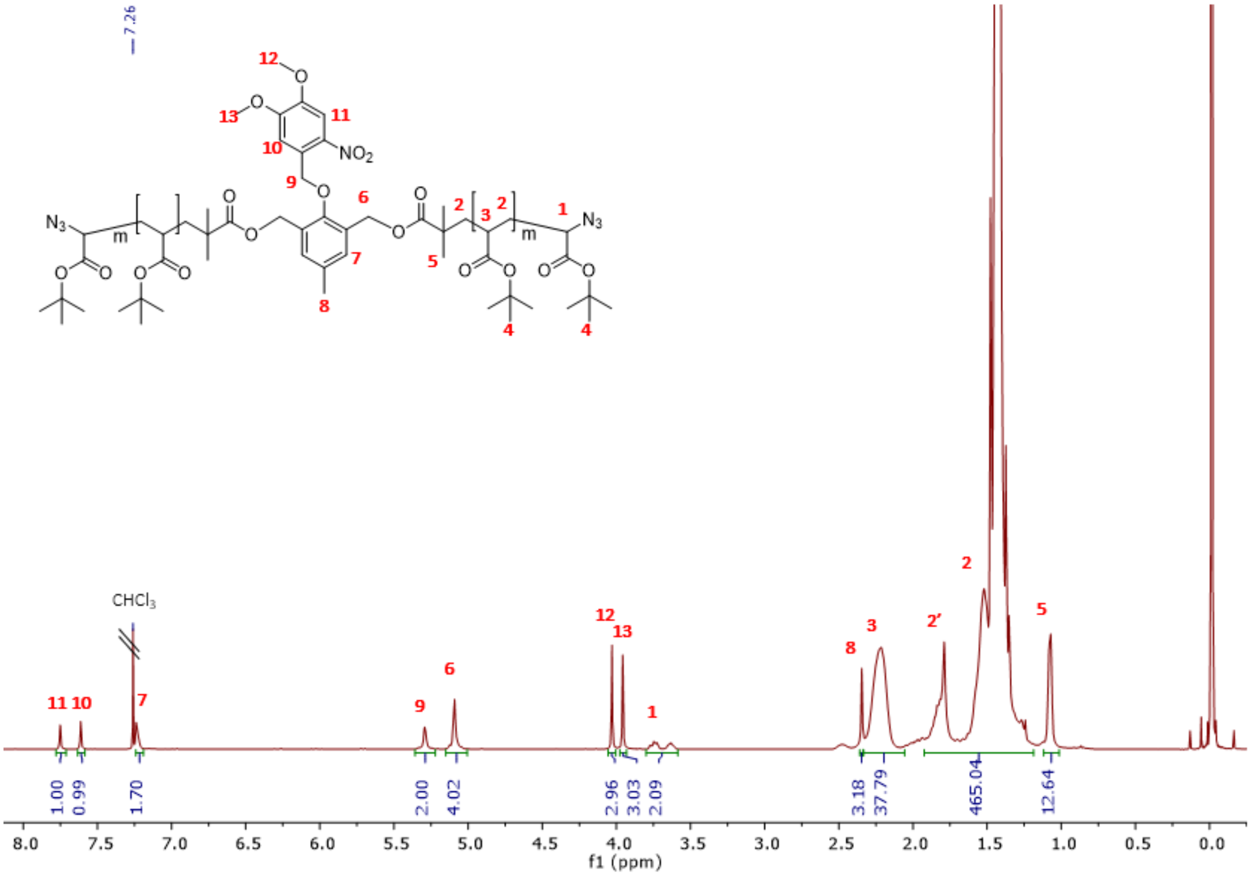

Figure S10: ^{1}H-NMR spectra of DMNB-PtBA-N_3.

C7-PtBA-N$_3$:

C7-PtBA-Br (450 mg, 0.07 mmol) and NaN_3 (91 mg, 1.40 mmol) were reacted according to the general procedure. The product was obtained as white solid in 91% yield (410 mg).

^{1}H NMR (400 MHz, Chloroform-*d*) δ 4.01 (t, *J* = 6.7 Hz, 4H, CH_2-C***H***$_2$-O-), 3.82 – 3.59 (m, 2H, -+ -C***H***-N_3), 2.39-2.09 (brs, 46H, PtBA backbone -C***H***-CO), 1.96-1.21 (m, 555H, O-CH_2-(C***H***$_2$)$_5$-CH_2-O + PtBA backbone –C***H***$_2$-CH- + -O-C(C***H***$_3$)$_3$), 1.13 (s, 12H, -CO-C-(C***H***$_3$)$_2$-PtBA). SEC (DMF + 25mM NH_4Ac): Mn = 4.8 kDa, Đ = 1.15, Expected Mn = 7.0 kDa.

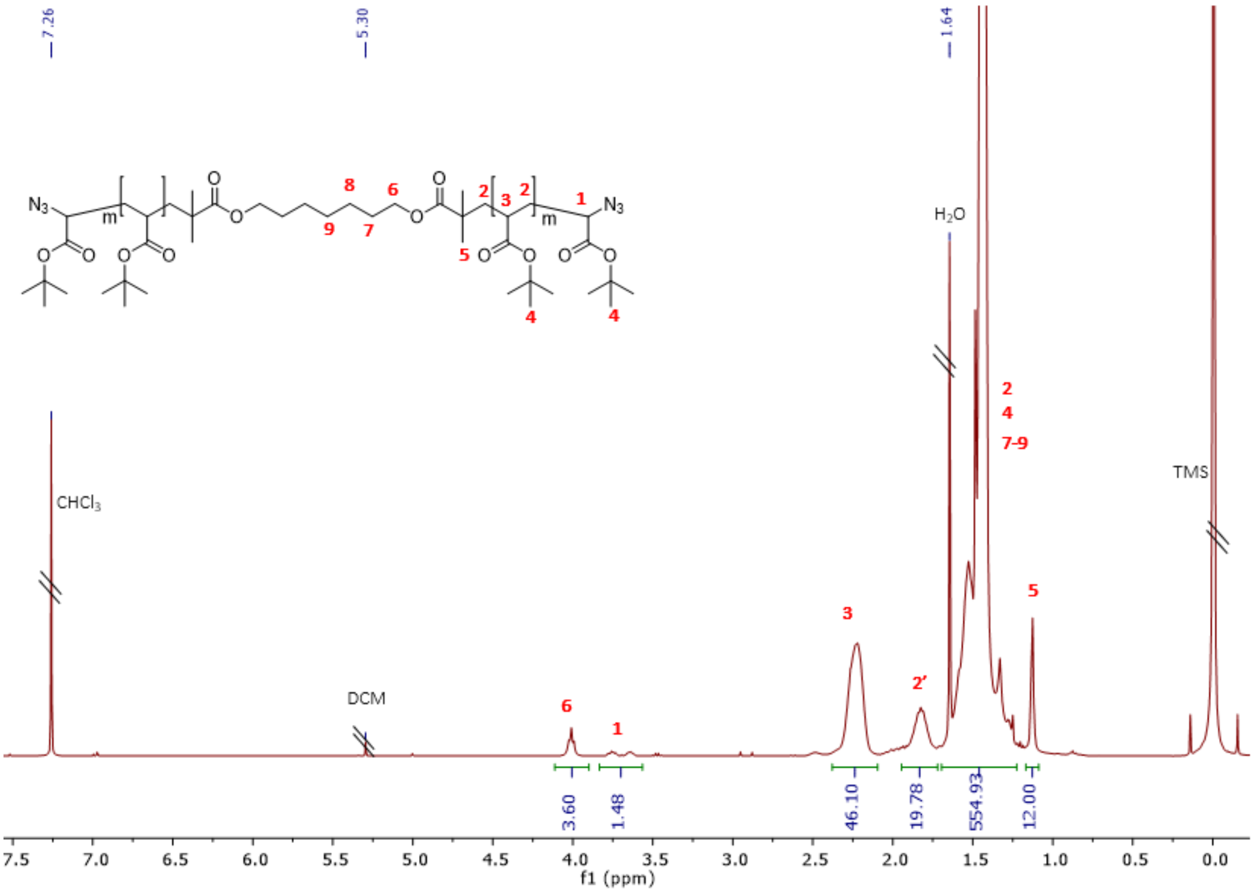

Figure S11: ^{1}*H-NMR spectra of C7-PtBA-N$_3$.*

Click reaction between di azide functionalized PtBA and esterase-responsive dendron

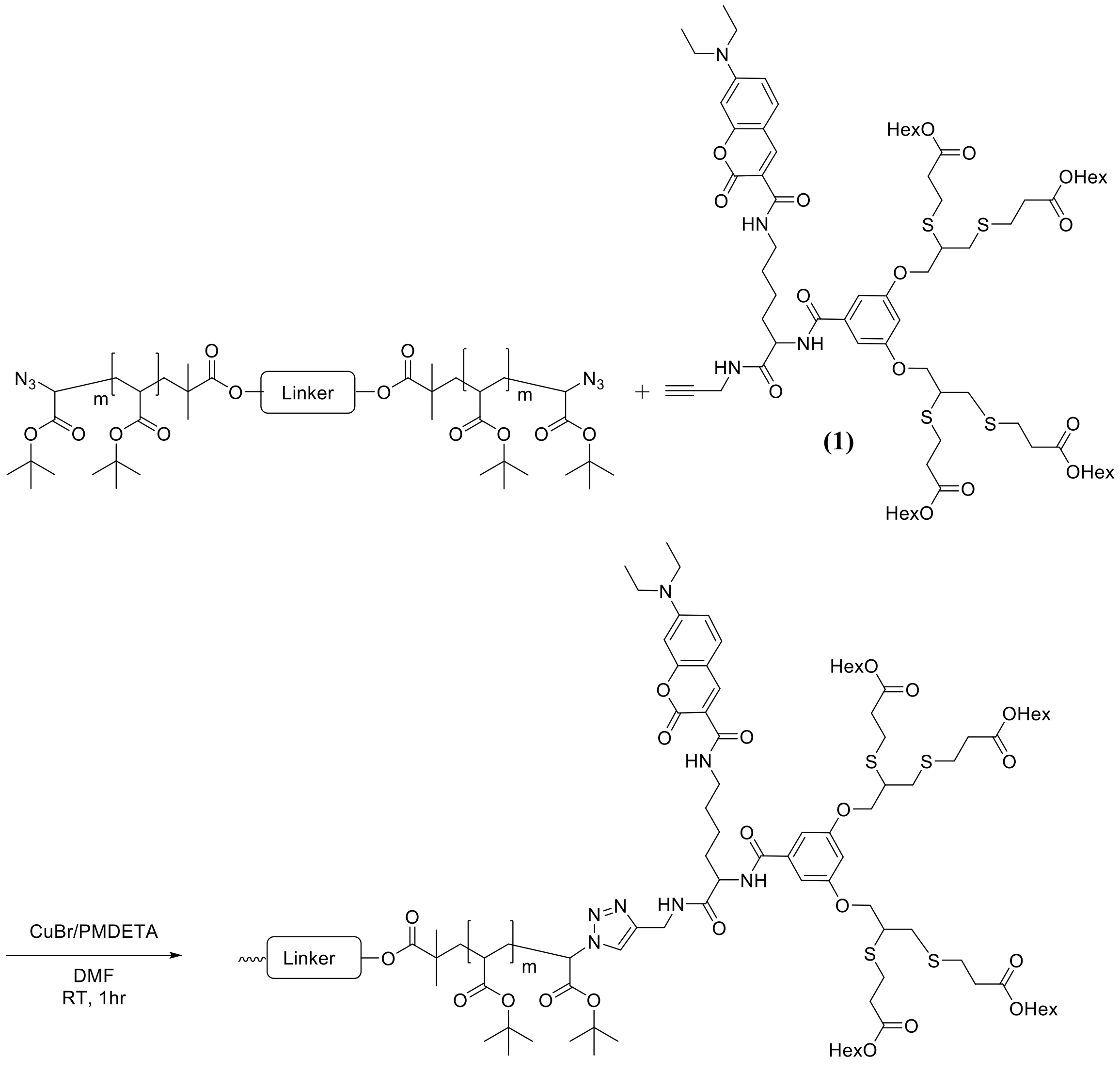

Figure S12: Synthetic scheme for the preparation of linker-PtBA-(D)-4xHex.

Dendron (1) - was synthesized as previously reported[2] and the spectroscopic characterization correlated well with these reports.

General procedure for CuAAC click reaction between di azide functionalized PtBA and dendron:

CuBr (3 eq. in respect to di azide functionalized PtBA) was loaded in 4 mL glass vial, which was sealed with a rubber septum. Vial was deoxygenated with three vacuum-nitrogen cycles and backfilled with nitrogen. In a separate 4 mL vial polymer-N_3 (1 eq.), dendron (2.6 eq.) and PMDETA (3 eq.) were dissolved in DMF (100 – 200 mg polymer/ mL) and purged with nitrogen for 2 minutes. The above-mentioned mixture was added into the CuBr containing vial using nitrogen flushed syringe and needle. Vial was thoroughly vortexed until clear green solution was obtained (approximately 30 seconds). Reaction was stirred at room temperature for 1 hour, filtered through syringe filter (0.44 µm, hydrophilic PTFE) and purified using LH20 (Sephadex®) size exclusion column and eluted with MeOH. Fractions that contained the product (identified by bright yellow color) were unified and MeOH was evaporated to dryness and product was dried on high vacuum. All polymers were obtained as bright yellow solids.

SS-PtBA-(D)-4xHex:

CuBr (7 mg, 0.048 mmol), SS-PtBA-N_3 (100 mg, 0.016 mmol), dendron (287 µL from 200 mg/ml solution in DMF, 0.041 mmol) and PMDETA (10 µL, 0.048 mmol) were reacted according to the general procedure. The product was obtained as yellow solid in 89% yield (130 mg).

^{1}H NMR (400 MHz, Chloroform-*d*): see following spectrum and assignments. SEC (DMF + 25mM NH_4Ac): Mn = 5.9 kDa, Đ = 1.20, Expected Mn = 9.1 kDa.

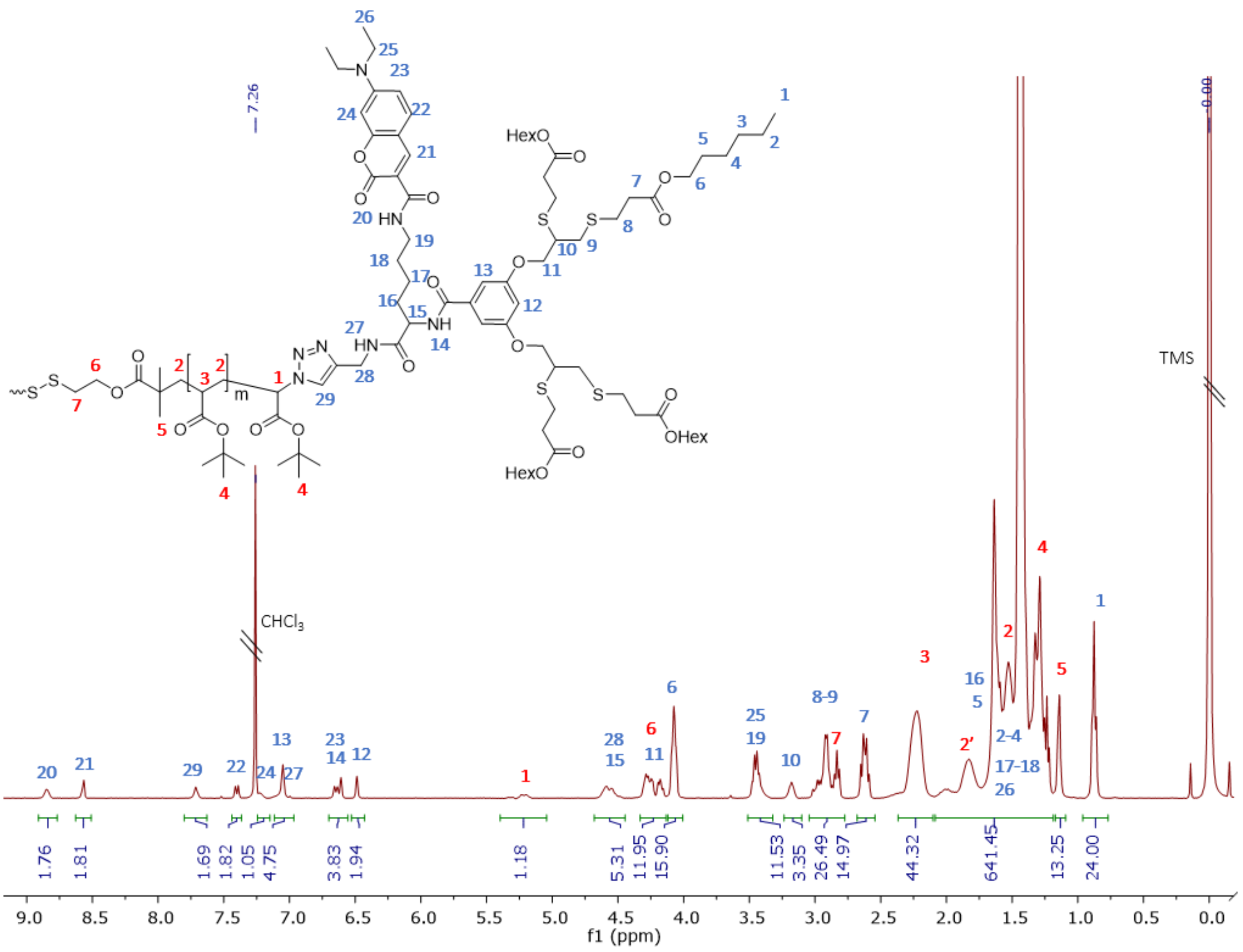

Figure S13: ^{1}H-NMR spectra of SS-PtBA-(D)-4xHex.

DMNB-PtBA-(D)-4xHex:

CuBr (6 mg, 0.042 mmol), DMNB-PtBA-N_3 (90 mg, 0.014 mmol), dendron (256 µL from 200 mg/ml solution in DMF, 0.036 mmol) and PMDETA (9 µL, 0.042 mmol) were reacted according to the general procedure. The product was obtained as yellow solid in 91% yield (130 mg).

^{1}H NMR (400 MHz, Chloroform-*d*): see following spectrum and assignments. SEC (DMF + 25mM NH_4Ac): Mn = 5.8 kDa, Đ = 1.24, Expected Mn = 9.2 kDa.

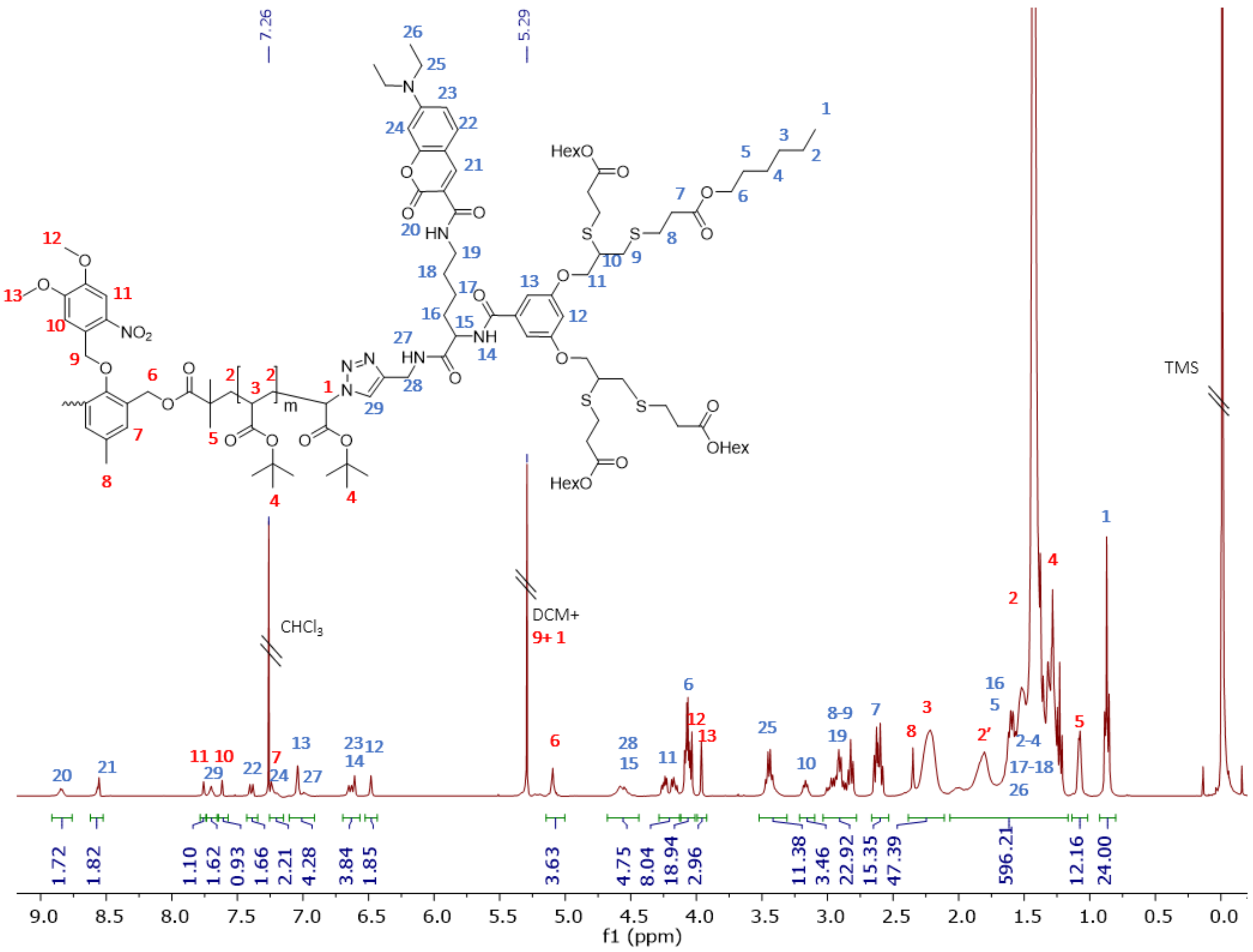

Figure S14: ^{1}H-NMR spectra of DMNB-PtBA-(D)-4xHex.

C7-PtBA-(D)-4xHex:

CuBr (7 mg, 0.048 mmol), C7-PtBA-N_3 (100 mg, 0.015 mmol), dendron (280 µL from 200 mg/ml solution in DMF, 0.040 mmol) and PMDETA (10 µL, 0.048 mmol) were reacted according to the general procedure. The product was obtained as yellow solid in 88% yield (123 mg).

^{1}H NMR (400 MHz, Chloroform-*d*): see following spectrum and assignments. SEC (DMF + 25mM NH_4Ac): Mn = 7.0 kDa, Đ = 1.19, Expected Mn = 9.8 kDa.

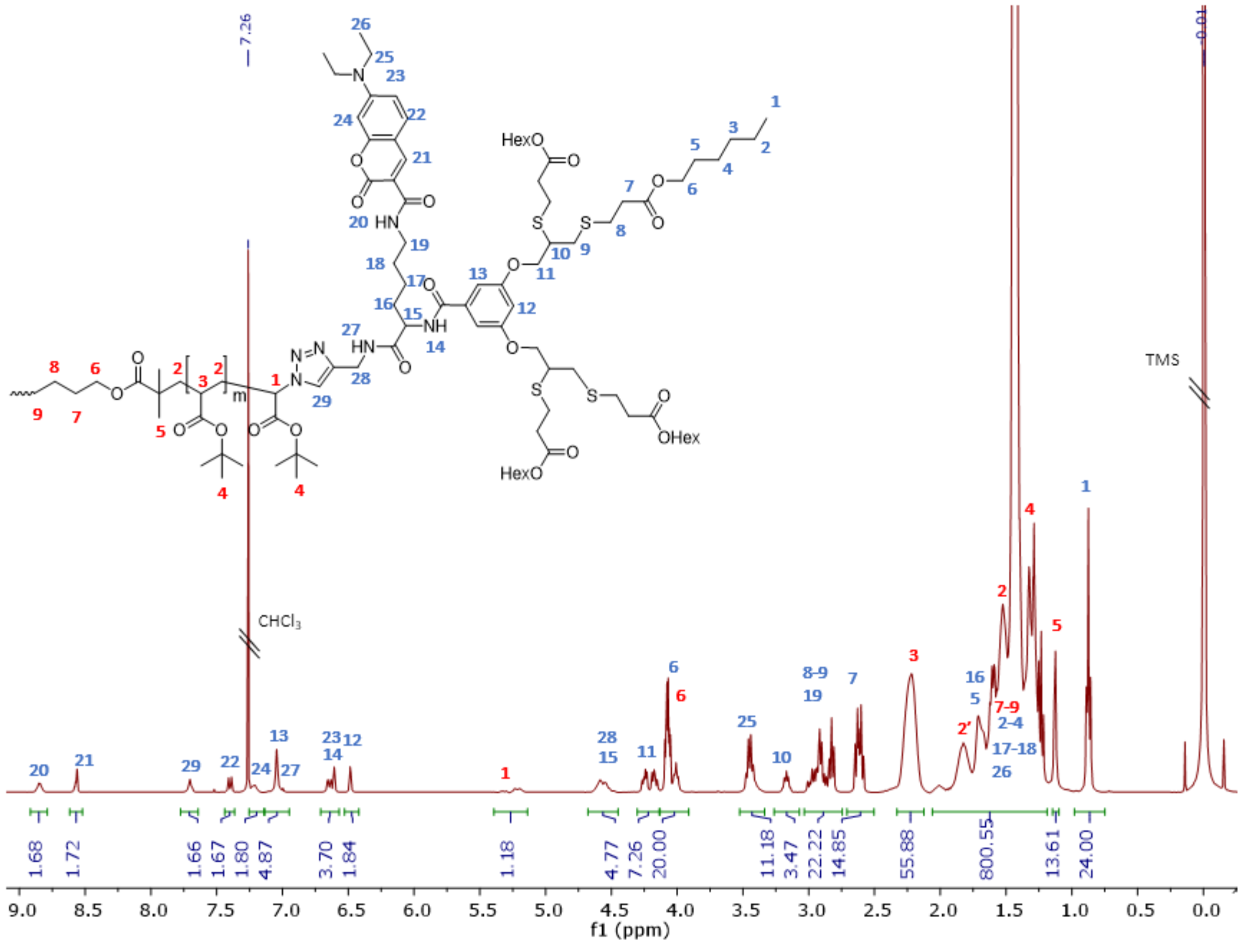

Figure S15: ^{1}H-NMR spectra of C7-PtBA-(D)-4xHex.

FT-IR measurements

IR spectra before and after CuAAC click reaction between di azide functionalized PtBA and dendron:

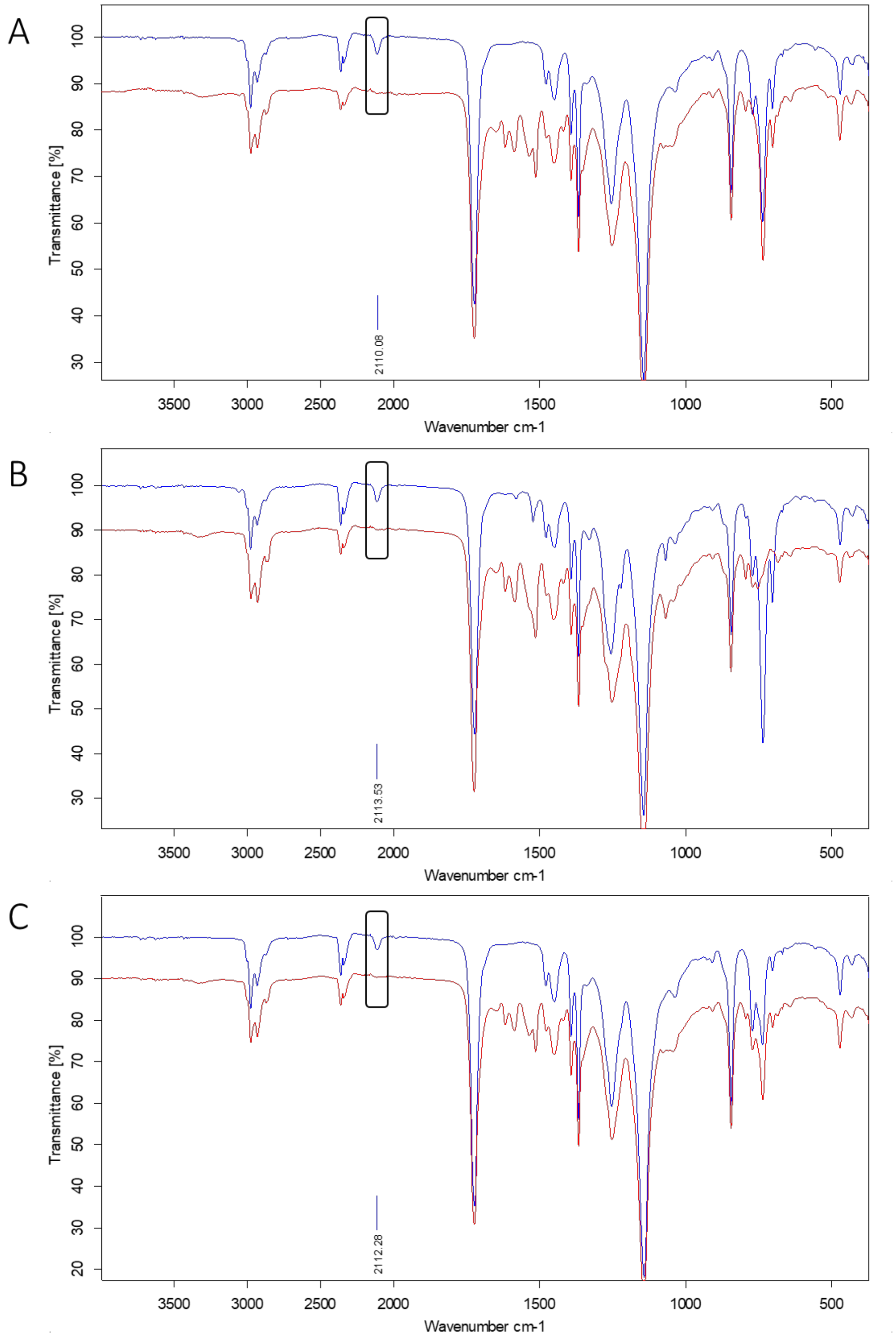

Figure S16: IR transmittance spectra of linker-PtBA-N3 (blue) and linker-PtBA-(D)-4xHex (red) show full disappearance of azide groups ($\tilde{\nu} \cong 2110$). A) SS linker, B) DMNB linker, C) C7 linker.

General procedure for tert-butyl deprotection of PtBA:

Tert butyl protection was removed by dissolving 100 mg of linker-PtBA-(D)-4xHex in 3 ml TFA and stirring at room temperature for one hour. TFA was removed under reduced pressure and further dried under high vacuum for 30 minutes. Product was redissolved in MeOH and further purified by LH20 SEC, to afford the final TBC amphiphiles (linker-PAA-(D)-4xHex). Due to the treatment with TFA, the TBC amphiphiles were partially splitted into DBC. All fractions were analyzed using HPLC (420 nm), and those with sufficient purity (above 95% TBC) were collected and used for further experiments and analysis.

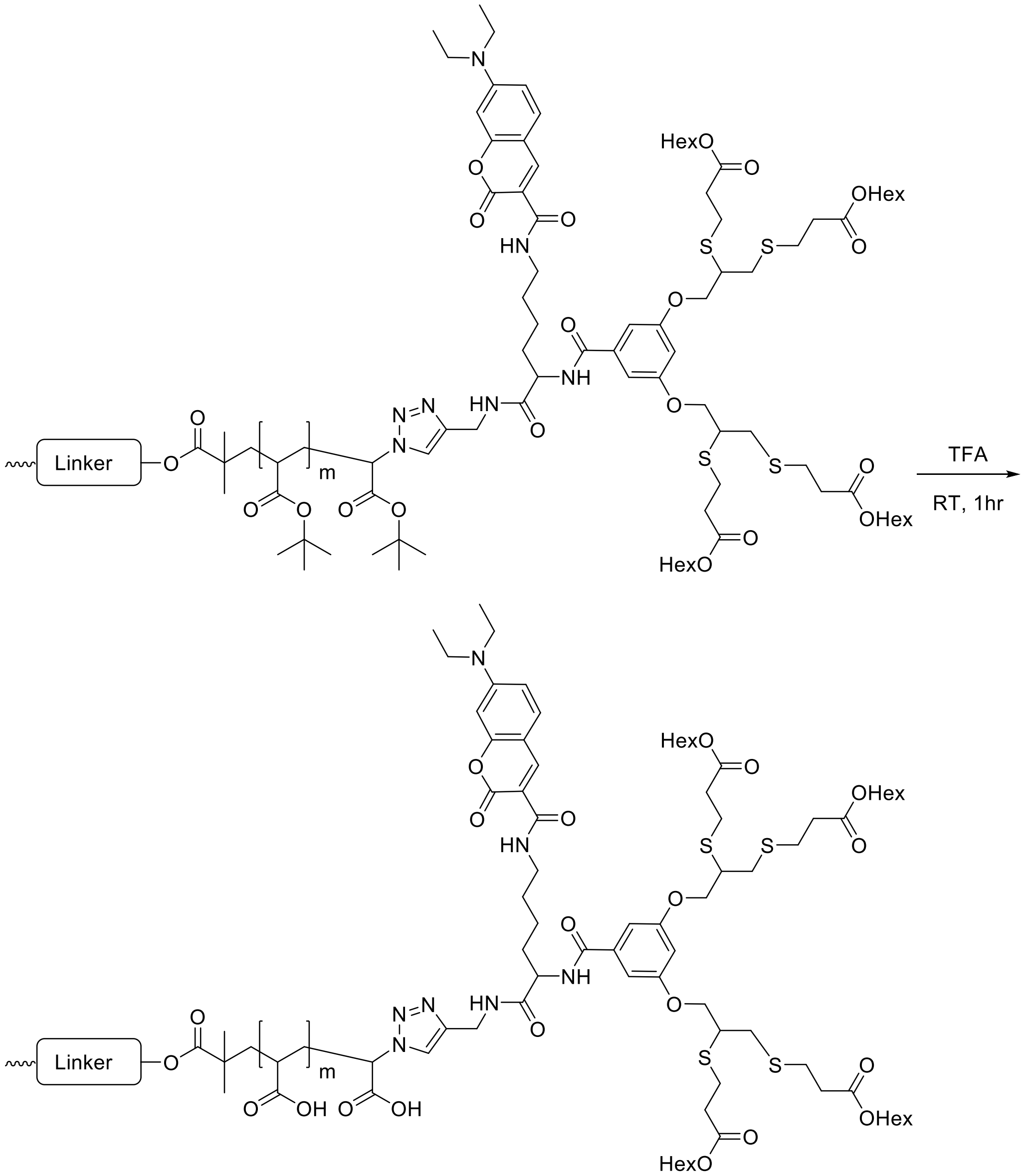

Figure S17: Synthetic scheme for the tert-butyl deprotection of linker-PtBA-(D)-4xHex.

SS-PAA-(D)-4xHex (SS-TBC):

SS-PtBA-(D)-4xHex was treated with TFA according to the general procedure.

^{1}H-NMR (400 MHz, CD_3OD): see following spectrum and assignments; ^{13}C-NMR (100 MHz, CD_3OD): δ 179.3, 178.4, 174.7, 173.6, 164.6, 160.9, 159.0, 154.5, 149.2, 137.2, 132.7, 111.7, 110. 1, 109.5, 107.7, 106.4, 97.4, 71.1, 66.0, 63.5, 55.3, 47. 1, 46.1, 42.8, 40.0, 37.9, 37.2, 36.2, 36.2, 35.8, 32.6, 30.0, 29.3, 28.4, 28.2, 26.7, 25.9, 24.3, 23.6, 14.5, 13.0; PAA polymers could not be analyzed by SEC due to the multiple acids interactions with the columns.

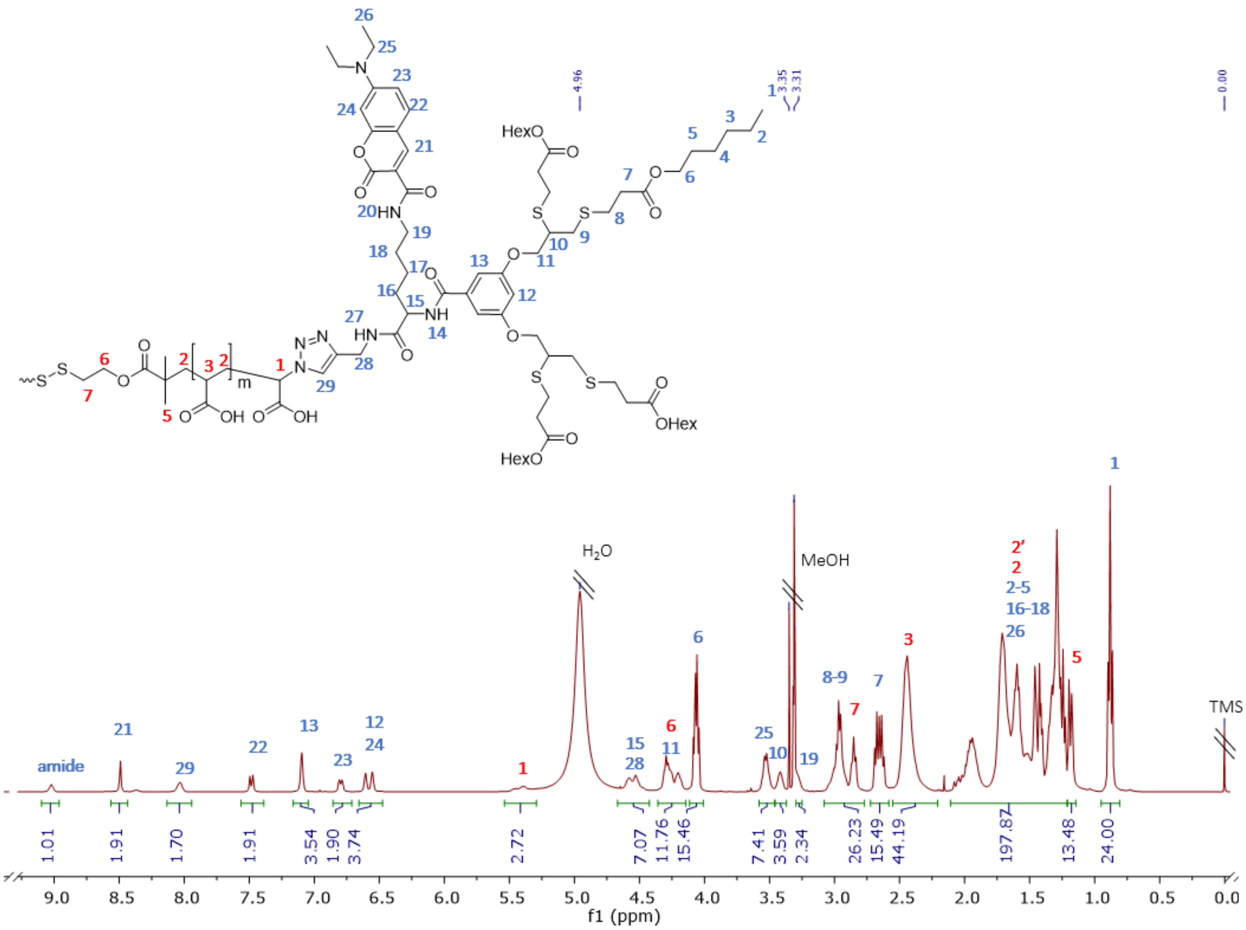

Figure S18: ^{1}H-NMR spectra of SS-PAA-(D)-4xHex.

DMNB-PAA-(D)-4xHex (DMNB-TBC):

DMNB-PtBA-(D)-4xHex was treated with TFA according to the general procedure.

^{1}H-NMR (400 MHz, CD_3OD): see following spectrum and assignments; ^{13}C-NMR (100 MHz, CD_3OD) δ 177.0, 172.2, 168.1, 163.9, 162.4, 159.4, 157.5, 153.0, 147.7, 135.7, 134.5, 132.0, 131.2, 129.2, 110.1, 109.1, 108.6, 108.0, 107.8, 106.2, 104.8, 95.9, 73.8, 69.6, 64.5, 61.3, 26.9, 26.5, 25.2, 24.1, 22.7, 22.1, 13.0, 11.4; PAA polymers could not be analyzed by SEC due to the multiple acids interactions with the columns.

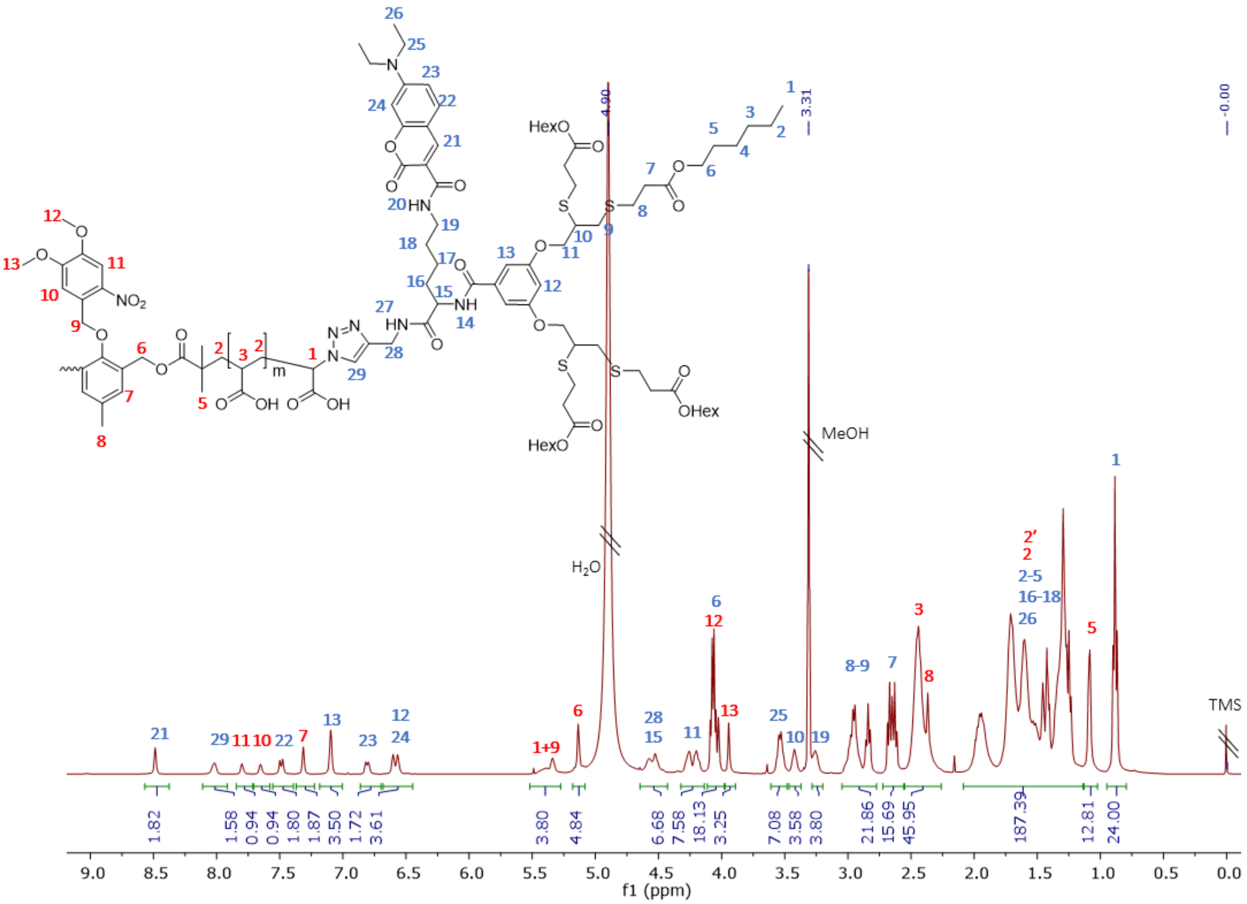

Figure S19: ^{1}H-NMR spectra of DMNB-PAA-(D)-4xHex.

C7-PAA-(D)-4xHex (C7-TBC):

C7-PtBA-(D)-4xHex was treated with TFA according to the general procedure.

^{1}H-NMR (400 MHz, CD_3OD): see following spectrum and assignments; ^{13}C-NMR (100 MHz, CD_3OD) δ 180.0, 178.6, 172.6, 167.7, 154.5, 149.1, 131.3, 121.8, 107.8, 71.2, 65.9, 46.7, 46.0, 42.7, 36.3, 32.6, 29.8, 26.9, 26.7, 23.6, 14.5, 12.9; PAA polymers could not be analyzed by SEC due to the multiple acids interactions with the columns.

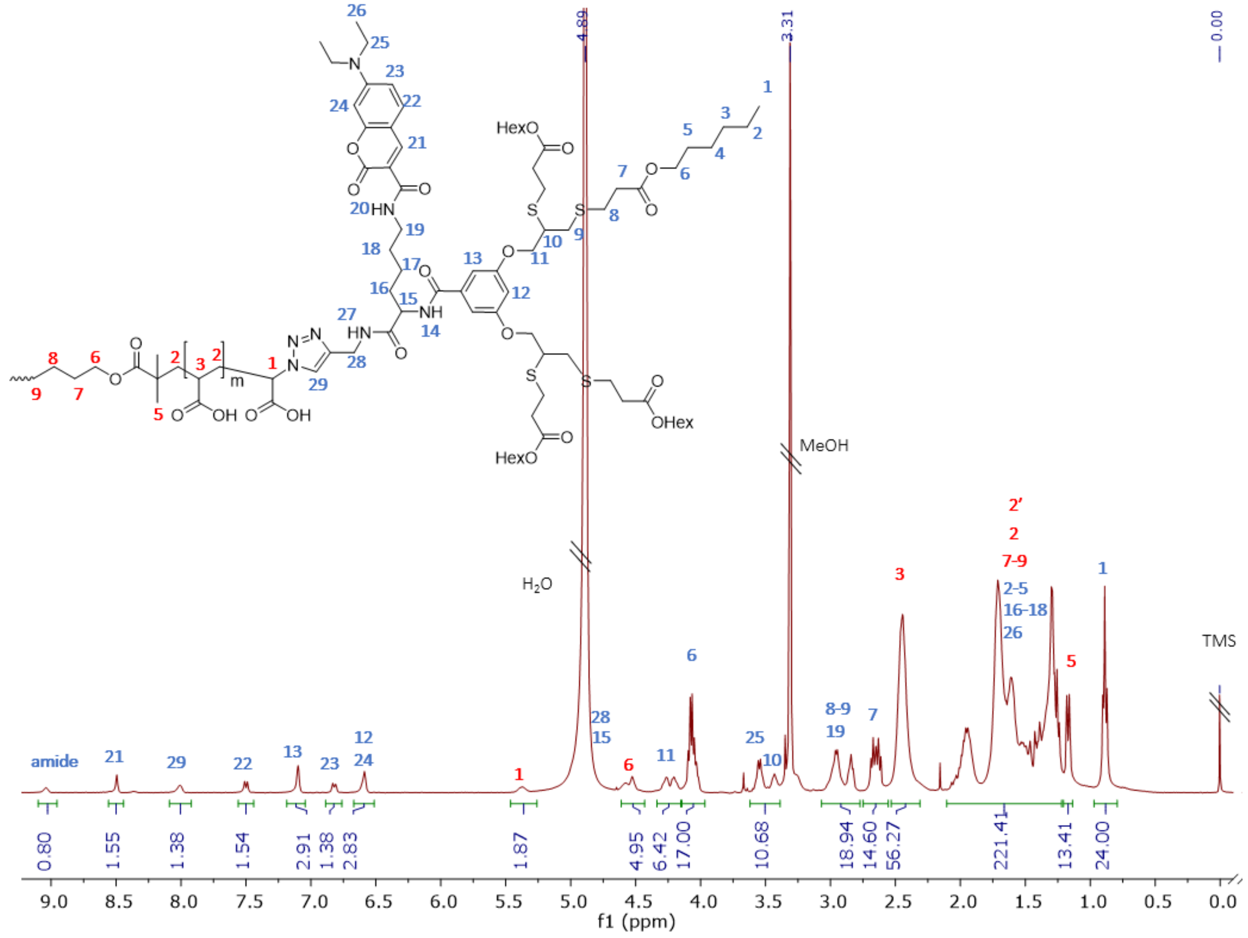

Figure S20: ^{1}H-NMR spectra of C7-PAA-(D)-4xHex.

Characterization of Polymer-dendron amphiphiles

HPLC measurements

Instrument: Waters Alliance e2695

Column: Aeris WIDEPORE, C4, 3.6 µm, 150x4.6 mm

Column temperature: 30°C

Sample temperature: 37°C

Solution A: 0.1% $HClO_4$:ACN 95:5v/v

Solution B: 0.1% $HClO_4$:ACN 5:95v/v

Solution C: ACN

Flow rate: 1ml/min

Gradient program for 30 minutes injection:

Time [minutes]	Sol. A [%]	Sol. B [%]	Sol. C [%]
0.0	95	0	5
1.0	95	0	5
20.0	0	95	5
23.0	0	95	5
23.1	95	0	5
30.0	95	0	5

Injection volume: 15 µL

Seal wash: H_2O:MeOH 90:10v/v

Needle wash: MeOH

Detector: Waters 2998 photodiode array detector

Sampling rate: 2 points/sec

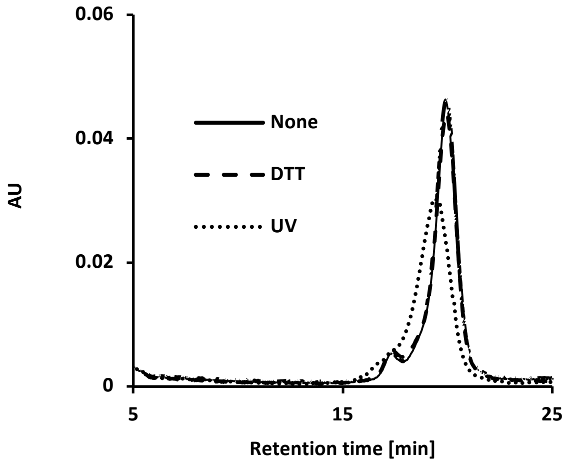

Figure S21: *HPLC chromatogram overlay (taken at 420 nm) of C7-TBC before (solid line) and after treatment with DTT (dashed line) or UV irradiation (dotted line).*

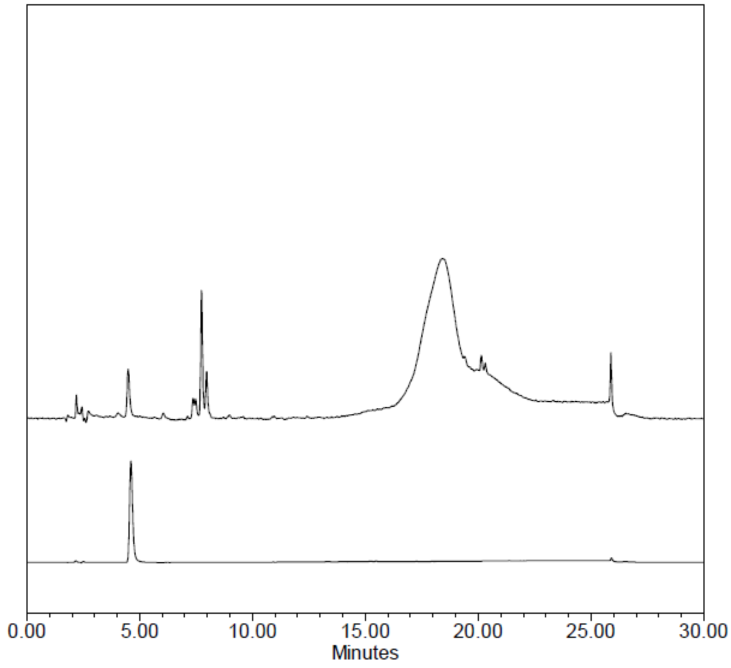

Figure S22: *HPLC chromatogram overlay (taken at 220 nm) of DMNB-TBC after UV irradiation (top) and 2,6-Bis(hydroxymethyl)-p-cresol (bottom).*

Size exclusion chromatography (SEC)

Instrument method:

Instrument: Malvern Viscotek GPCmax

Columns: 2xPSS GRAM 1000Å + PSS GRAM 30Å

Columns temperature: 50°C

Flow rate: 0.5 mL/min

Injection time: 90 min

Injection volume: 50 µL from a 10 mg/ml sample

Diluent + mobile phase: DMF + 25mM NH_4Ac

Needle wash: DMF

Sample preparation: The Polymer-dendron amphiphiles were directly dissolved in the diluent to give a final concentration of 10 mg/mL and filtered with 0.45 µM PTFE filter.

Splitting of SS- and DMNB-PtBA-(D)-4xHex

SS-PtBA-(D)-4xHex sample was treated with DTT (20 mM), incubated for 30 minutes and was analyzed by SEC. DMNB-PtBA-(D)-4xHex sample was irradiated with UV light for 30 minutes, and water and Et_3N were added (50 µL and 20 µL, respectively, for 1 mL) and sample was incubated for 3 hours prior to injection.

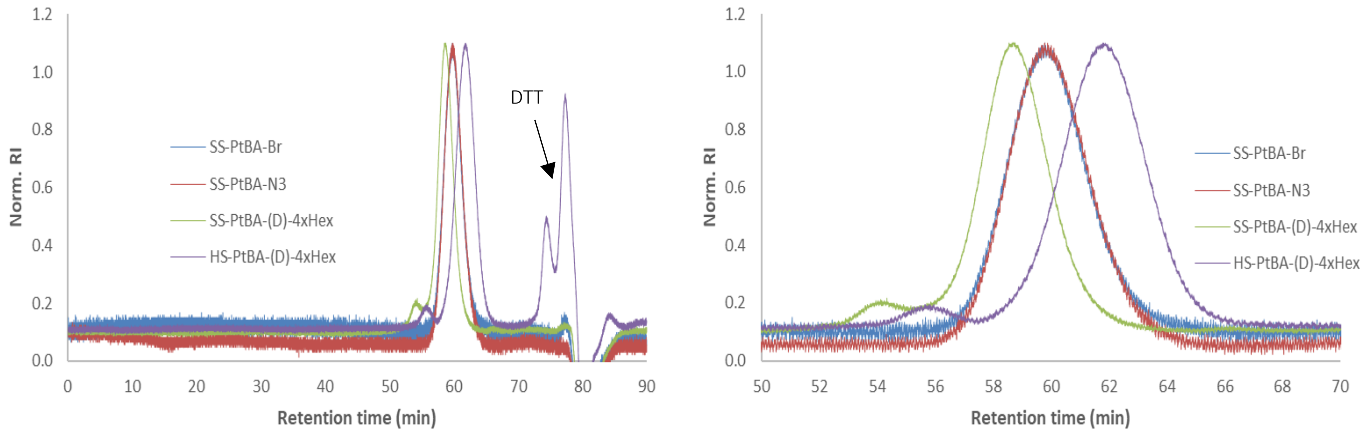

<u>Figure S23:</u> SEC traces overlay of SS-PtBA-Br, SS-PtBA-N_3, SS-PtBA-(D)-4xHex (TBC) and SH-PtBA-(D)-4xHex (DBC, after exposure to DTT). Mn values for SS-PtBA-(D)-4xHex (TBC): 5.9 kDa ; SH-PtBA-(D)-4xHex (DBC): 2.7 kDa.

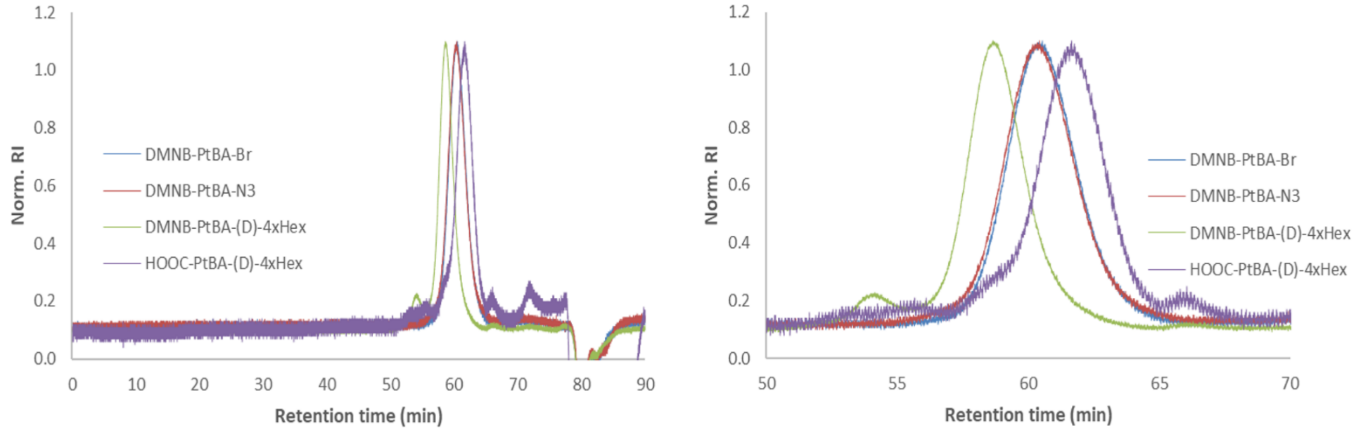

<u>Figure S24:</u> SEC traces overlay of DMNB-PtBA-Br, DMNB-PtBA-N_3, DMNB-PtBA-(D)-4xHex (TBC) and HO_2C-PtBA-(D)-4xHex (DBC, after exposure to UV radiation). Mn values for DMNB-PtBA-(D)-4xHex (TBC): 5.8 kDa; HO_2C-PtBA-(D)-4xHex (DBC): 2.9 kDa.

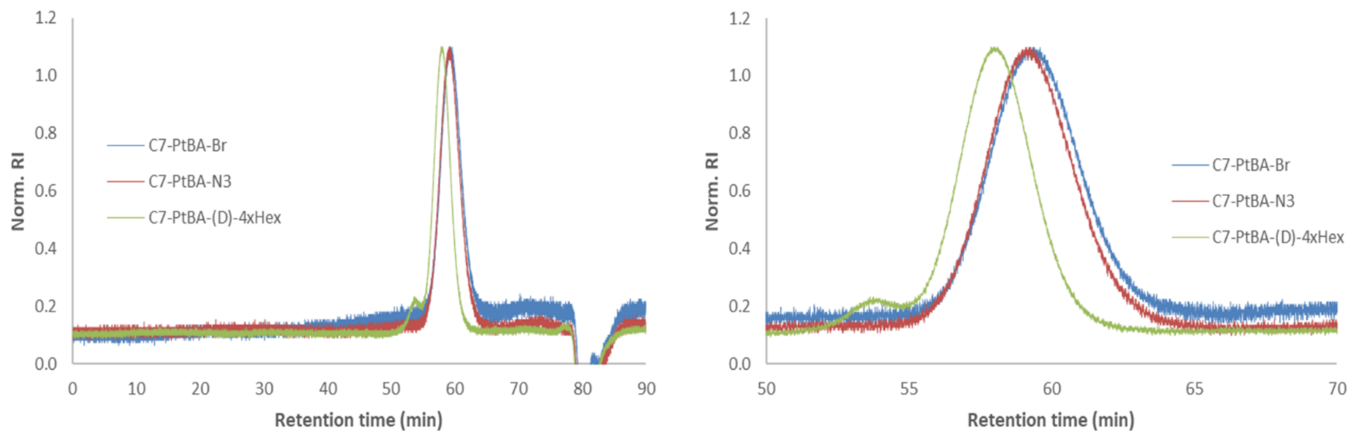

<u>Figure S25:</u> SEC traces overlay of C7-PtBA-Br, C7-PtBA-N_3, C7-PtBA-(D)-4xHex (C7 TBC).

Splitting of TBC amphiphiles

Activation of SS-TBC:

A micellar solution of SS-TBC amphiphile was prepared by directly adding phosphate buffer saline pH 7.4 to solid polymer to a final concentration of 80 μM. Vial was vortexed until full solubility was obtained and then placed in an ultrasonic bath for 15 minutes. Then, the solution was treated with DTT (20 μL from 1M solution in PBS into 1 mL, to yield final DTT concentration of 20 mM), and was incubated for 0.5 hours at 37°C. The transformation from TBC into DBC amphiphiles was followed at 37°C by monitoring the area under the peak of the TBC and DBC amphiphiles by HPLC at 420 nm.

Activation of DMNB-TBC:

A micellar solution of the DMNB-TBC amphiphile was prepared by directly adding phosphate buffer saline pH 7.4 to solid polymer to a final concentration of 80 μM. Vial was vortexed until full solubility was obtained and then placed in an ultrasonic bath for 15 minutes. Then, the solution was irradiated with UV light (365 nm) for 30 min, and was further incubated for 2.5 hours at 37°C. The transformation from TBC into DBC amphiphiles was followed at 37°C by monitoring the area under the peak of the TBC and DBC amphiphiles by HPLC at 420nm.

Critical micelles' concentration (CMC)

General procedure of measurement:

Preparation of diluent:

Nile Red stock solution (2500 µM in ethanol) was diluted into a phosphate buffer saline (137 mM NaCl, 10 mM phosphate, 2.7 mM KCl; pH 7.4) to afford a final concentration of 1.5 µM.

Preparation and measurement of samples:

The Polymer-dendron amphiphile was directly dissolved in the diluent to give a final concentration of 250 µM. Solution was vortexed vigorously until the amphiphile completely dissolved and further sonicated for 15 minutes in an ultrasonic bath. This solution was divided into two samples, one of which was treated according to the general procedure for splitting of the TBC amphiphiles into DBCs. Each sample was consecutively diluted by a factor of 1.5 with the diluent to afford a series of 24 samples with decreasing concentration. 150 µL of each sample were loaded onto a 96 well plate and a fluorescence emission scan was performed for each well. In order to determine the amphiphile's CMC – the maximum emission of Nile Red (at about 630 nm) was plotted versus the amphiphile's concentration. This procedure was repeated trice for each amphiphile and mean value is reported as the CMC value and the standard deviation as measurement error.

Instrument method:

Instrument: TECAN Infinite M200Pro

Excitation: 550 nm

Emission intensity scan: 580-800 nm

Step: 5 nm

Number of flashes: 15

Gain: 100

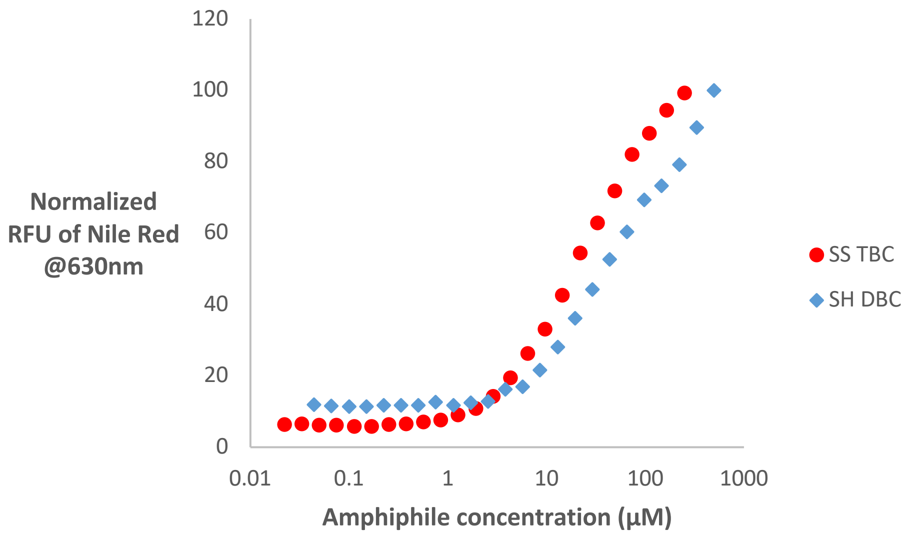

Figure S26: CMC measurement of SS-TBC and SH-DBC. CMC values for SS TBC: 3±1µM, for SH-DBC: 5±1µM.

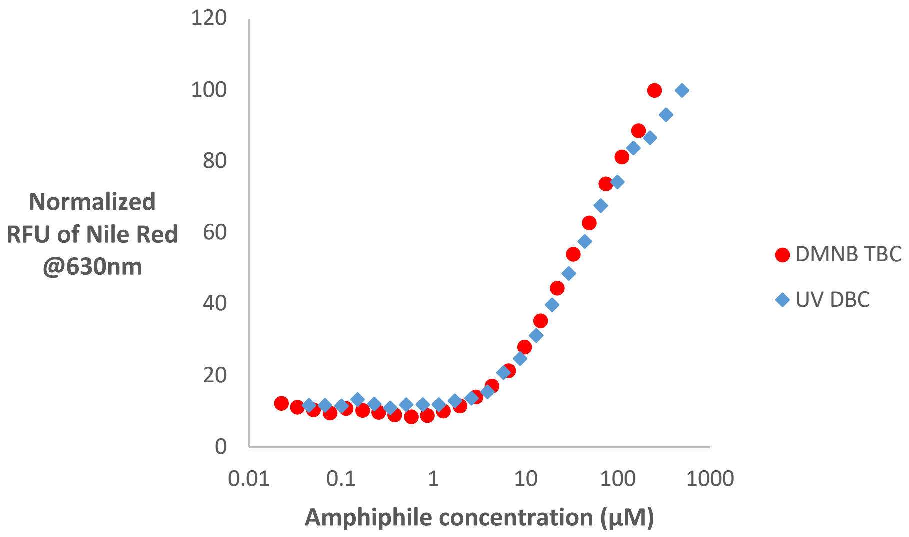

Figure S27: CMC measurement of DMNB-TBC and UV-DBC. CMC values for DMNB-TBC: 3±1µM, for UV-DBC: 4±1µM.

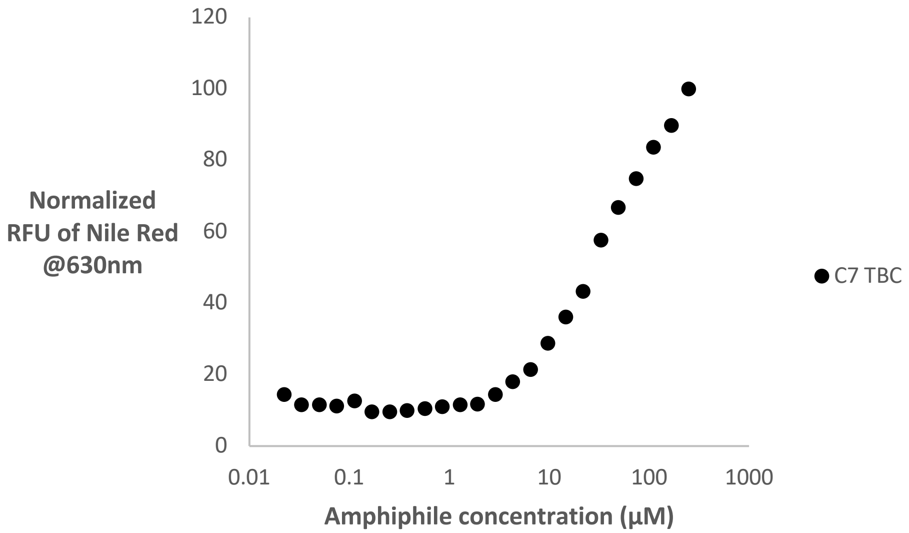

<u>Figure S28</u>: CMC measurement of C7-TBC. CMC value for C7-TBC: 4±1µM.

TEM imaging

Sample preparation:

The amphiphiles were dissolved in phosphate buffer saline (pH 7.4) to afford a final concentration of 80 μM. 30 μL of the hybrid solution were deposited onto carbon coated copper grids. The excessive solvent of the droplet was wiped away using a solvent-absorbing filter paper after 1 minute and the sample grids were left to dry in air at RT for 8 hours. Then, grids were inspected in transmission electron microscope (TEM), operated at 120 kV (JEM-1400Plus).

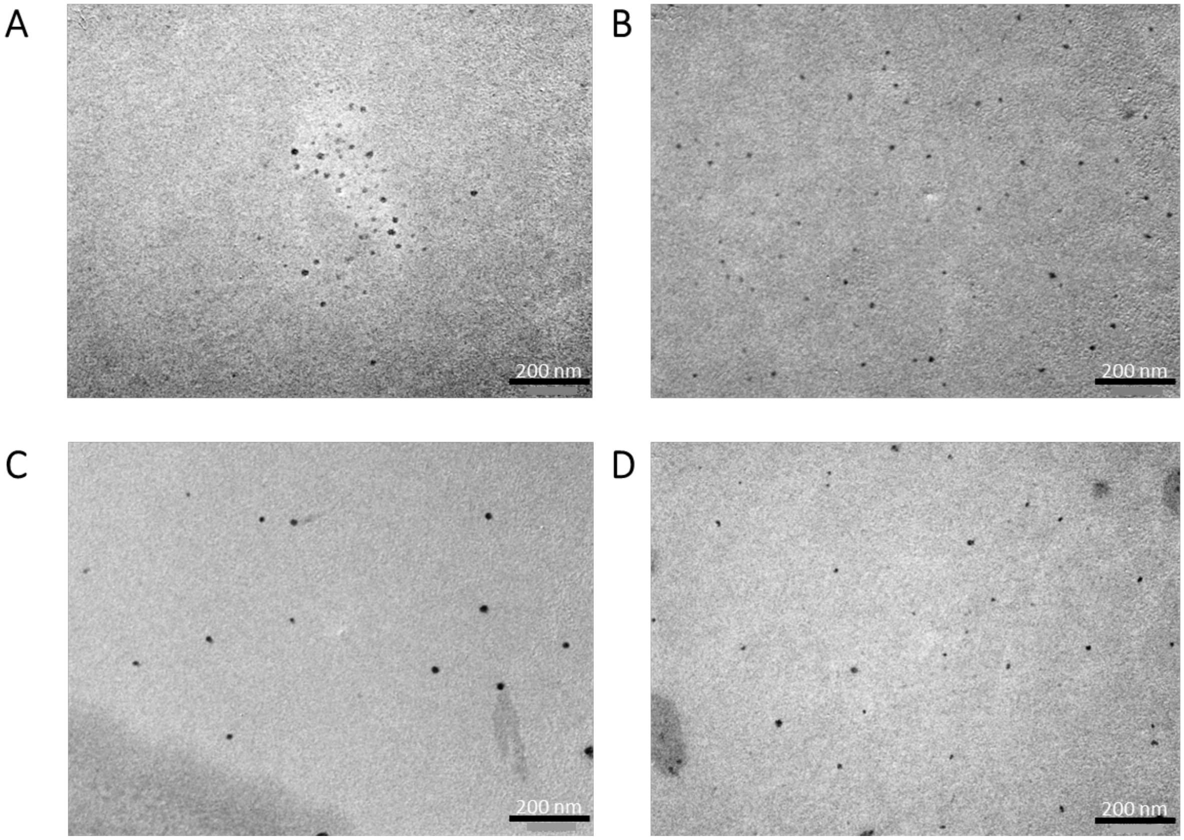

Figure S29: TEM images of micellar solution of SS-TBC (A), DMNB-TBC (B), SH-DBC (C), UV-DBC (D). Average diameter (for 10 particles or more): (A) 13±2nm ; (B) 12±2nm ; (C) 13±2nm ; (D) 12±2nm.

Enzymatic degradation experiments:

A micellar solution of the tested TBC amphiphile was prepared by directly adding phosphate buffer saline pH 7.4 to solid polymer to a final concentration of 80 µM. Vial was vortexed until full solubility was obtained and then placed in an ultrasonic bath for 15 minutes. For DBC micelles, the TBC amphiphile solution was treated according to general procedure for splitting. PLE or PBS were added (20 µL into 1 mL, to yield final PLE concentration of 0.1 µM) and degradation was followed at 37°C either by monitoring the area under the peak of the parent amphiphile by HPLC or the emission at 560 nm by fluorimeter. Each experiment was conducted trice and the reported values in each time point are the mean valued and the standard deviation is the error.

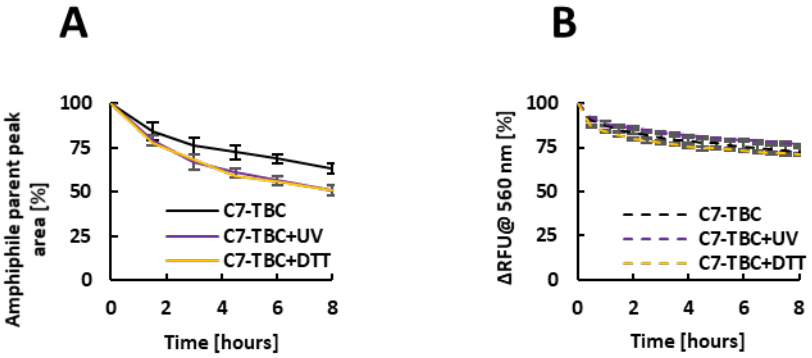

Figure S30: *Enzyme responsiveness of C7-TBC after treatment with DTT (yellow line), exposure to UV light (purple line) or PBS (black line). Enzymatic degradation profiles as obtained by HPLC (A; solid lines) and fluorescence spectroscopy (B; dashed lines).*

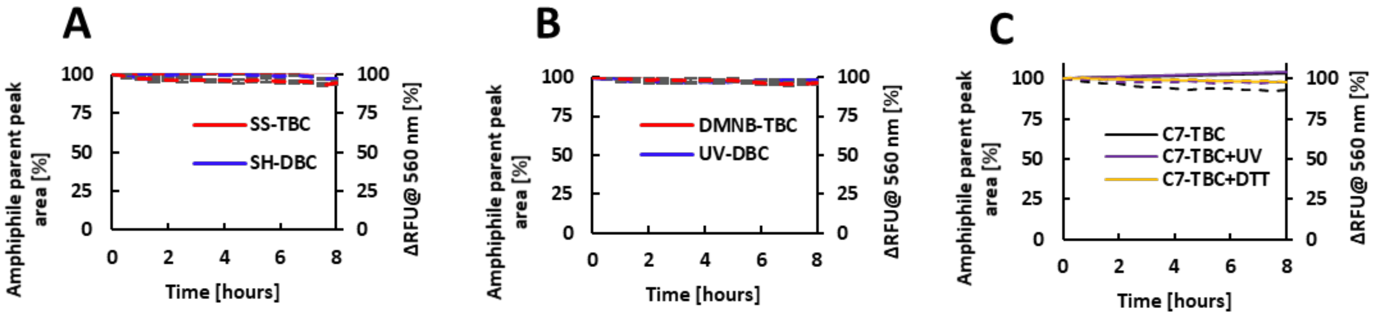

Figure S31: *Control experiments of Disulfide (A), DMNB (B) and C7 (C) amphiphiles in the absence of PLE, after treatment with DTT, exposure to UV radiation or PBS. Amphiphiles' stability profiles as obtained by HPLC (solid lines) and fluorescence spectroscopy (dashed lines).*

Dynamic light scattering

All samples for DLS measurements were prepared as mentioned before, and were filtered prior to measurement using 0.45 µm nylon filter.

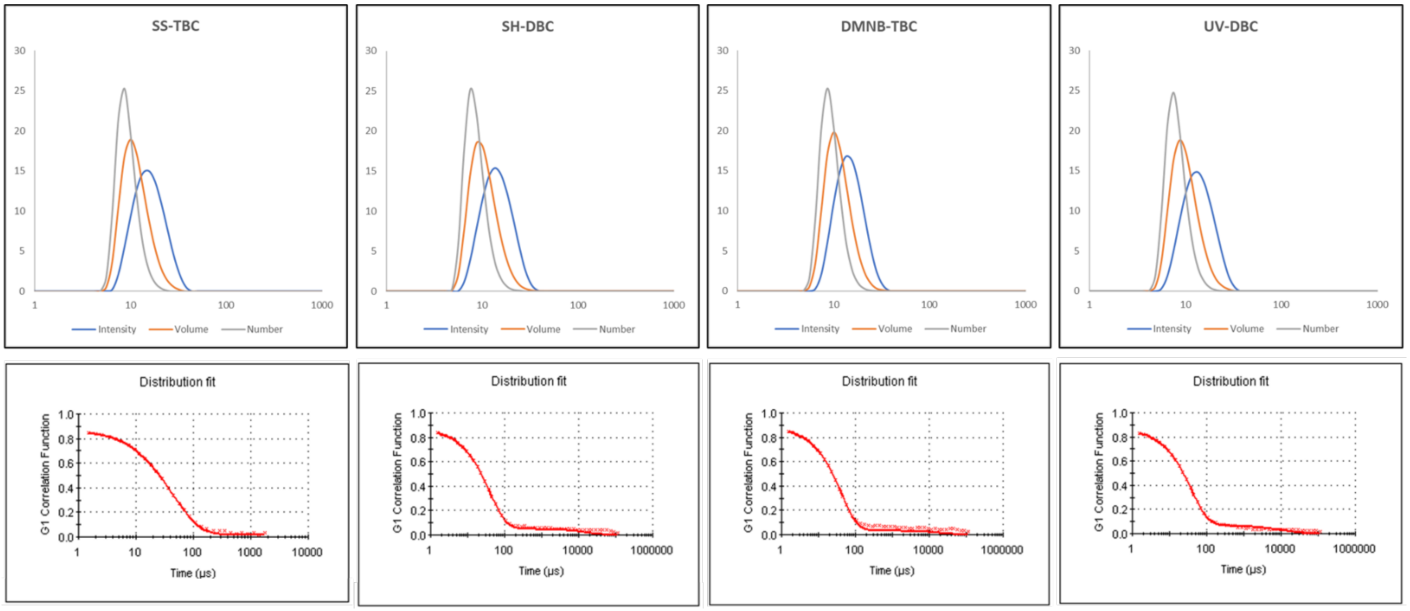

Figure S32: DLS measurements raw data for TBC-amphiphiles before and after activation. [TBC] = 80 µM, [DTT] = 20 mM.

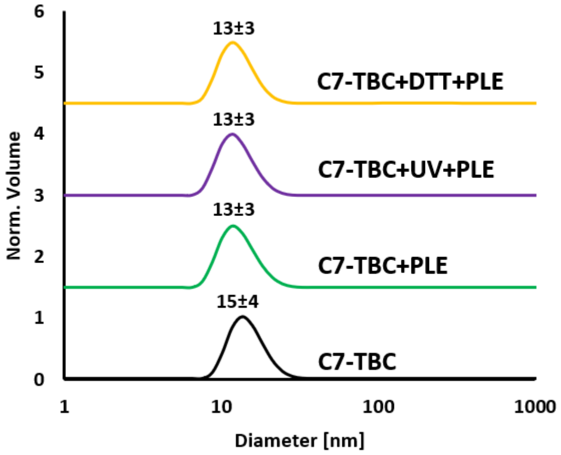

Figure S33: DLS measurements of C7-TBC after 8 hours incubation upon the addition of PBS (black line) or PLE, in addition to prior treatment with DTT (yellow line), exposure to UV radiation (purple line) or PBS (green line). [TBC] = 80 µM, [PLE] = 0.1 µM, [DTT] = 20 mM.

Nile red release experiments:

Nile Red stock solution (2500 µM in ethanol) was diluted into a phosphate buffer saline pH 7.4 to afford a final concentration of 1.5 µM. This solution was used to dissolve the solid TBC amphiphile to a final amphiphile concentration of 80 µM. Vial was vortexed until full solubility was obtained and then placed in an ultrasonic bath for 15 minutes. This solution was divided into two samples, one of which was treated according to the general procedure for splitting of the TBC amphiphiles into DBCs. TBC and DBC micellar solutions were treated with PLE or PBS as described in the 'enzymatic degradation experiments section'. Nile red release profile was obtained by monitoring the maximum emission of Nile Red (at about 630 nm) by fluorimeter at 37°C. Each experiment was conducted trice and the reported value in each time point is the mean value and the standard deviation is the error.

Micellar stability in the presence of BSA:

Micellar solution of the tested TBC amphiphile was prepared in PBS ([TBC] = 80 µM, DBC solution was prepared by treating the TBC solution with DTT or UV-irradiation and ensuring full splitting by HPLC). Then, into 450 µL of the above solution, 50 µL of either BSA solution (55 mg/ml in PBS) or PBS were added, and solution was vortexed to obtain final concentrations of 72 µM for amphiphile and 5.5 mg/ml for BSA. Emission spectra was recorded every 15 minutes for 2 hours (λEx = 420 nm).

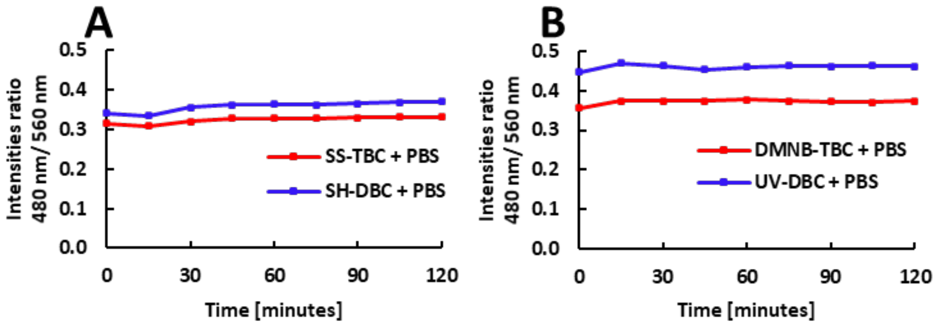

Figure S34: Unimer/ micelle fluorescence intensities ratio (480nm/560nm) over time for (A) SS-TBC and SH-DBC, (B) DMNB-TBC and UV-BDC in PBS; [TBC] = 72 µM, λEx = 420 nm.

References:

[1] L. Peles-Strahl, R. Sasson, G. Slor, N. Edelstein-Pardo, A. Dahan, R. J. Amir, *Macromolecules* **2019**, *52*, 3268–3277.

[2] G. Slor, A. R. Olea, S. Pujals, A. Tigrine, V. R. D. La Rosa, R. Hoogenboom, L. Albertazzi, R. J. Amir, *Biomacromolecules* **2021**, *22*, 1197–1210.
